# A Comprehensive Review of Traditional Medicinal Uses, Geographical Distribution, Botanical Characterization, Phytochemistry, and Pharmacology of *Aralia continentalis* Kitag.

**DOI:** 10.3390/molecules29153529

**Published:** 2024-07-26

**Authors:** Luyun Zhang, Huri Piao, Hao Zang

**Affiliations:** 1 Key Laboratory of Natural Medicines of the Changbai Mountain, Ministry of Education, Yanbian University, Yanji 133002, China; m18843581583@163.com (L.Z.); piaohr@ybu.edu.cn (H.P.); 2 School of Pharmacy and Medicine, Tonghua Normal University, Tonghua 134002, China

**Keywords:** *Aralia continentalis* Kitag., chemical composition, geographical distribution, botanical characterization, pharmacological effects

## Abstract

*Aralia continentalis* Kitag. (*A. continentalis*) holds significant medicinal value among the *Aralia* genus. It has traditionally been employed in ethnomedicine to address a wide range of conditions, including wind–cold–dampness arthralgia; rheumatic pain in the waist and lower extremities; lumbar muscular strain; injuries resulting from falls, fractures, contusions, and strains; headache; toothache; and abscesses. Modern pharmacological research has validated its therapeutic potential, encompassing anti-inflammatory, analgesic, antioxidant, antimicrobial, insecticidal, hepatoprotective, anti-diabetic, and cytotoxic properties, among other pharmacological effects. To compile comprehensive knowledge on *A. continentalis*, a rigorous literature search was undertaken utilizing databases like SciFinder, PubMed, and Web of Science. This review seeks to delve into the plant’s traditional applications, geographical distribution, botanical characteristics, phytochemistry, and pharmacology. The objective is to lay a foundation and propose novel research directions for exploring the plant’s potential applications. Currently, one hundred and fifty-nine compounds have been isolated and identified from *A. continentalis*, encompassing diterpenoids, steroids, triterpenoids, volatile components, phenolics, vitamins, trace elements, and other compounds. Notably, diterpenoids, steroids, triterpenoids, volatile components, and phenolics have exhibited pronounced pharmacological effects, such as anti-inflammatory, analgesic, antioxidant, hepatoprotective, antidiabetic, and antimicrobial activities. However, despite the extensive research conducted, further studies are imperative to unravel new components and mechanisms of action, necessitating more in-depth investigations. This comprehensive exploration could pave the way for advancing and harnessing the potential of *A. continentalis*.

## 1. Introduction

**Figure 1:**
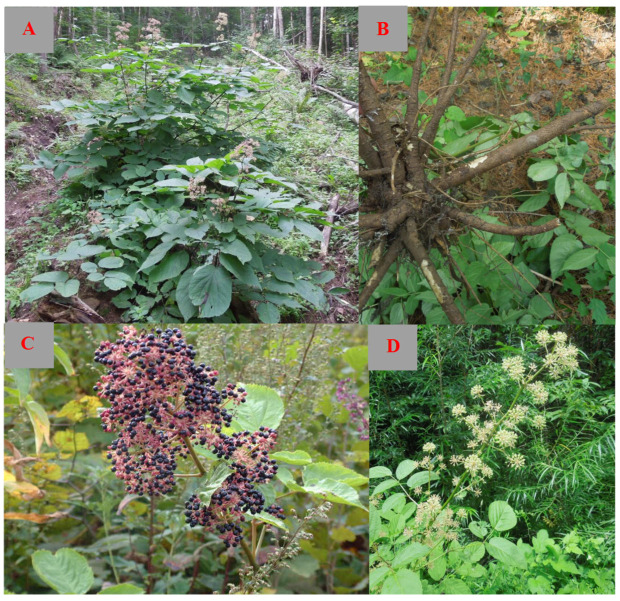
Morphology of *Aralia continentalis*: aboveground part (**A**), root (**B**), fruit (**C**), and flower (**D**).

*Aralia continentalis* Kitag. (*A. continentalis*) (Figure 1) is a significant plant in the *Aralia* genus, widely utilized in folk medicine and as a wild vegetable in spring. Identified synonymously as Aralia cordata var. continentalis (Kitag.) Y. C. Zhu, various active components have been isolated and identified from *A. continentalis*, including diterpenoids [1,2,3,4,5,6,7,8,9,10,11,12,13,14,15,16], steroids and triterpenoids [15,16,17,18,19,20,21,22,23], volatile components [24,25,26], phenolics [9,27,28,29], vitamins and trace elements [30,31], and other compounds [1,9,32,33]. Studies have shown that extracts and isolated compounds from *A. continentalis* possess diverse anti-inflammatory and analgesic [34,35,36,37,38,39,40,41,42,43,44,45,46,47,48,49,50,51,52,53,54,55,56,57,58,59], antioxidant [60,61,62,63,64,65,66,67,68,69,70,71,72,73,74,75], antimicrobial [76,77,78,79,80,81,82,83,84,85], insecticidal [86,87,88], hepatoprotective [89,90,91,92], anti-diabetic [93,94,95,96], and cytotoxic properties [97,98,99,100,101,102,103], among other pharmacological effects [104,105,106,107,108], with a particular focus on its anti-inflammatory and analgesic qualities. The traditional Korean medicine, Insampaedok-san, containing *A. continentalis*, has been historically used for cold-related symptoms, showing effects such as analgesia and antipyresis [109].

With the growing interest in natural remedies, studying the chemical composition and pharmacological effects of *A. continentalis*, a plant with medicinal properties, becomes essential. Despite the existing research that has summarized the phytochemistry and pharmacology of *A. continentalis* [110], there are still notable gaps in its coverage. These gaps encompass the incomplete classification of components, a limited listing of constituents, and a dearth of information regarding the chemical structure, precise theoretical molecular weight, and characterization methods for these components. Moreover, the mechanisms underpinning the pharmacological effects are often inadequately described and clarified. In contrast, our review endeavors to address these shortcomings by presenting a comprehensive list of one hundred and fifty-nine components, accompanied by structural information for each compound, including its name, formula, exact theoretical molecular weight, characterization method, references, and source. Furthermore, our review introduces a novel classification of pharmacological research, distinct from previous reports. Critically, we incorporate the most recent research findings on *A. continentalis*, providing an up-to-date and comprehensive perspective on this topic. Hence, the purpose of our review is to fill these gaps by offering a thorough evaluation of the traditional medicinal applications, geographical distribution, botanical characterization, phytochemistry, and pharmacology of *A. continentalis*. By systematically examining the existing research on *A. continentalis*, this review provides a robust foundation for medicinal research and suggests new avenues for researchers, fostering its progress and application in contemporary medicine.

In order to ensure the reliability and integrity of the information gathered for this review, a comprehensive data collection process was carried out using different databases, including SciFinder, PubMed, Web of Science, ProQuest, CNKI, and KCI. The search encompassed a comprehensive range of sources, including peer-reviewed journals, Ph.D. dissertations, master’s theses, conference papers, and seminal works on Chinese herbal medicines. To broaden the spectrum of this research, specific keywords such as phytochemistry, diterpenoids, geographical distribution, pharmacology, biological activity, antioxidant, antibacterial, anti-inflammatory, cytotoxicity, and other relevant terms were utilized, along with the term *A. continentalis*. This approach enabled the retrieval of a diverse array of pertinent studies published from 1954 to June 2024.

## 2. Traditional Medicinal Uses

*A. continentalis*, known as Changbaisongmu in Chinese and Dokwhal in Korean, has been widely utilized in traditional medicine from ancient times to now. The roots and root bark are used as medicine, typically harvested after autumn. The roots are either dried fresh or sun-dried before use. The plant is characterized as warm in nature, with a pungent and bitter flavor. Its primary functions include dispelling wind, eliminating dampness, promoting blood circulation, relieving pain, and detoxification [111]. It is commonly used to address conditions such as wind–cold–dampness arthralgia; rheumatic pain in the waist and lower extremities; lumbar muscular strain; injuries from falls, fractures, contusions, and strains; headache; toothache; and abscesses. It is not recommended for patients with yin deficiency, internal heat, or wind–heat common cold. When taken internally, it is commonly boiled with water in a dosage ranging from 3 to 10 g or steeped in Chinese Baijiu. For external use, a suitable quantity can be used for washing or external application [111]. Specific prescriptions involving *A. continentalis* include decocting *A. continentalis* and *Clematis chinensis* to treat wind–cold–dampness arthralgia and pain in the loin and knee, applying crushed *A. continentalis* to the knee for knee pain, and decocting *A. continentalis*, *Nepeta cataria*, and scallion for treating acute sprains or strains of hands and feet [111].

## 3. Geographical Distribution

**Figure 2:**
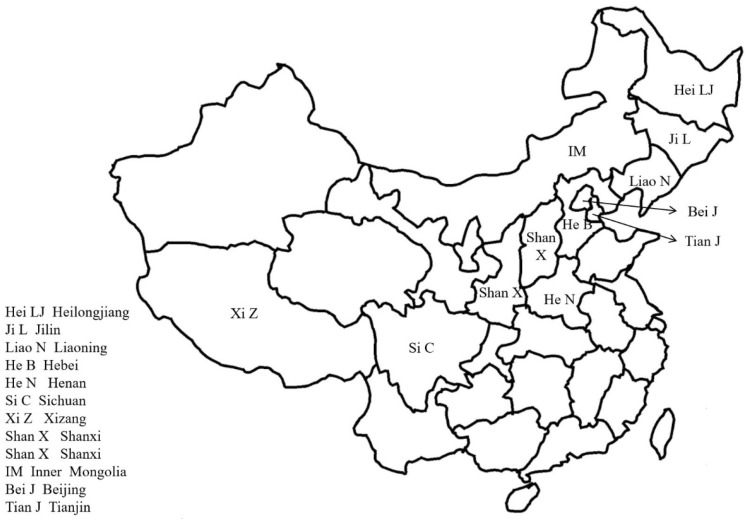
The general geographical distribution of *A. continentalis* in China.

*A. continentalis* is distributed across various regions in China, including Jilin, Liaoning, Heilongjiang, Beijing, Tianjin, Hebei, Shanxi, Shanxi, Inner Mongolia, Henan, Sichuan, and Xizang. Figure 2 provides a visual representation of its general geographical distribution within the country. Additionally, it can be found in North Korea, South Korea, and Russia [111,112]. This plant thrives beneath the trees in forests and on grassy slopes, typically at altitudes ranging from 800 to 3200 m [112].

## 4. Botanical Characterization

This plant is a perennial herb with blocky, thick rhizomes underground. The aboveground stem can reach up to 1 m in height, adorned with grey, fine hairs on its upper part. The leaves are two or three pinnate compound leaves, with petioles measuring 11.5–24.5 cm long and sparsely covered in grey, fine hairs. The stipules and petiole base are connate, ovate, or narrowly ovate and 2.5–6 mm long, featuring irregular teeth on the upper part and dense, grey, fine hairs on the outside. The pinna has 3–7 leaflets, which are membranous. The top leaflets are inverted or elliptical, while the side leaflets are elongated or elliptical to oval in shape. The leaflets either lack petioles or have petioles as long as 1 cm. The panicle is large, up to 55 cm long, terminal or axillary, and densely branched, with grey, fine hairs on the main axis and branches. The umbel has a diameter of 1.5–2 cm with numerous flowers. The peduncle’s total length ranges from 1 to 2 cm and is hairy, with ovate bracts that have pointed apices, membranous margins, ciliate edges, and a length of 1.5 to 2 mm. The pedicel is characterized by its short and sturdy nature, measuring 5 to 6 mm in length; it is hairy and surrounded by lanceolate bracteoles approximately 1 mm long. The calyx, devoid of hair, is 1.5 mm long and possesses five sharp triangular teeth along the edge. There are five triangular-ovate petals, each 2 mm long; five stamens measuring 2.5 mm; an ovary with five chambers; and a style with five parts fused at the base and free at the apex. The fruit, which is purple-black and has five ridges, is around 3 mm in diameter, with a persistent style roughly 2 mm long that is fused below the middle, free at the top, and retroflexed. The plant typically blossoms from July to August and bears fruit from August to September [112].

## 5. Phytochemistry

Upon preliminary chemical analysis, it was discovered that *A. continentalis* primarily comprises glucosides, with notable exclusions being those of the cardiac-stimulant variety and saponins. Furthermore, alkaloids were present in minimal, trace quantities, predominantly in their reduced form. Additionally, the plant was found to contain volatile oils [113], enhancing its chemical complexity. The roots, stems, and leaves of *A. continentalis* were further subjected to extraction and qualitative analysis using water, ethanol, and petroleum ether. The findings indicated that *A. continentalis* harbors a diverse array of bioactive components, including saponins, flavonoids, alkaloids, coumarins, and others. Notably, its leaves and roots are abundant in total saponins and flavonoids [114]. This preliminary study establishes a foundation for the further development and utilization of the effective bioactive components present in *A. continentalis*. In recent years, research on the components of *A. continentalis* has gained momentum. According to reports, one hundred and fifty-nine compounds have been isolated or identified from *A. continentalis*, belonging to six distinct categories: diterpenoids, steroids and triterpenoids, volatile compounds, phenolic compounds, vitamins and trace elements, and other compounds. This remarkable abundance of bioactive ingredients highlights the promising potential of *A. continentalis* as a valuable resource for drug development.

### 5.1. Diterpenoids

**Figure 3:**
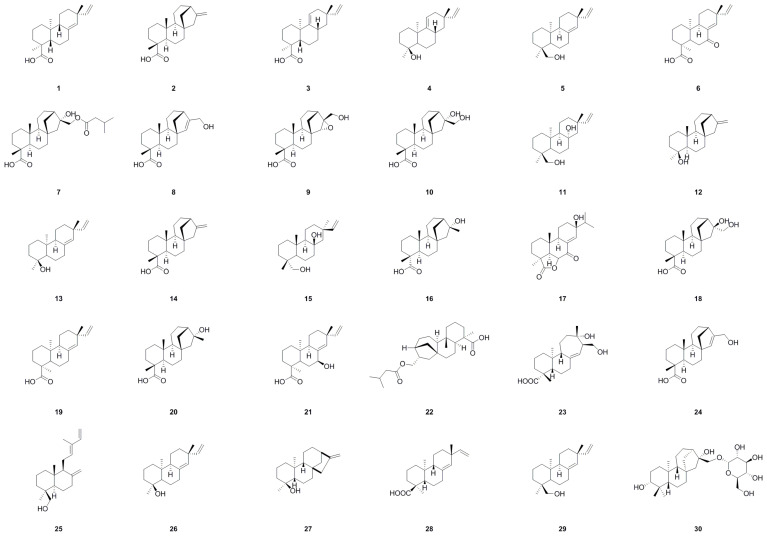
The chemical structures of diterpenoids from *Aralia continentalis* were accurately depicted using ChemDraw Professional 15.0 software.

Currently, diterpenoids are typically found in the genus *Aralia* [115], serving in particular as distinctive components of *A. continentalis*. A total of thirty diterpenoid components (**1**–**30**), each possessing unique structural features and pharmacological properties, have been successfully isolated or identified (Table 1, Figure 3). This renders them an intriguing subject for ongoing scientific investigation.

### 5.2. Steroids and Triterpenoids

**Figure 4:**
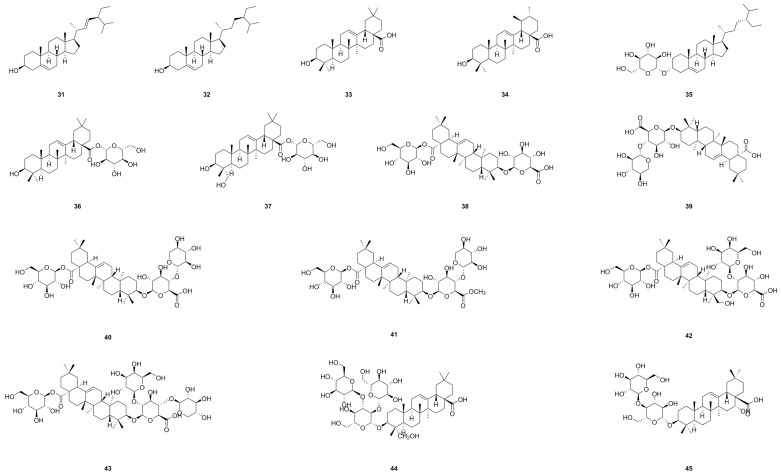
The chemical structures of steroids and triterpenoids from *Aralia continentalis* were accurately depicted using ChemDraw Professional 15.0 software.

The genus *Aralia* is abundant in steroids and triterpenoids [115]. Currently, *A. continentalis* has yielded fifteen steroids and triterpenoids (**31**–**45**), as detailed in Table 2 and Figure 4. Each of these compounds represents distinct opportunities for further exploration and pharmacological research. Stigmasterol (**31**), which was extracted from *A. continentalis*, has shown significant inhibitory effects on the growth, acid production, adhesion, and synthesis of water-insoluble glucan by *S. mutans* [17]. These discoveries indicate that stigmasterol may effectively counteract the cariogenic properties of *S. mutans*, thereby justifying the traditional practice of utilizing *A. continentalis* extracts for treating dental ailments among local populations.

### 5.3. Volatile Components

**Figure 5:**
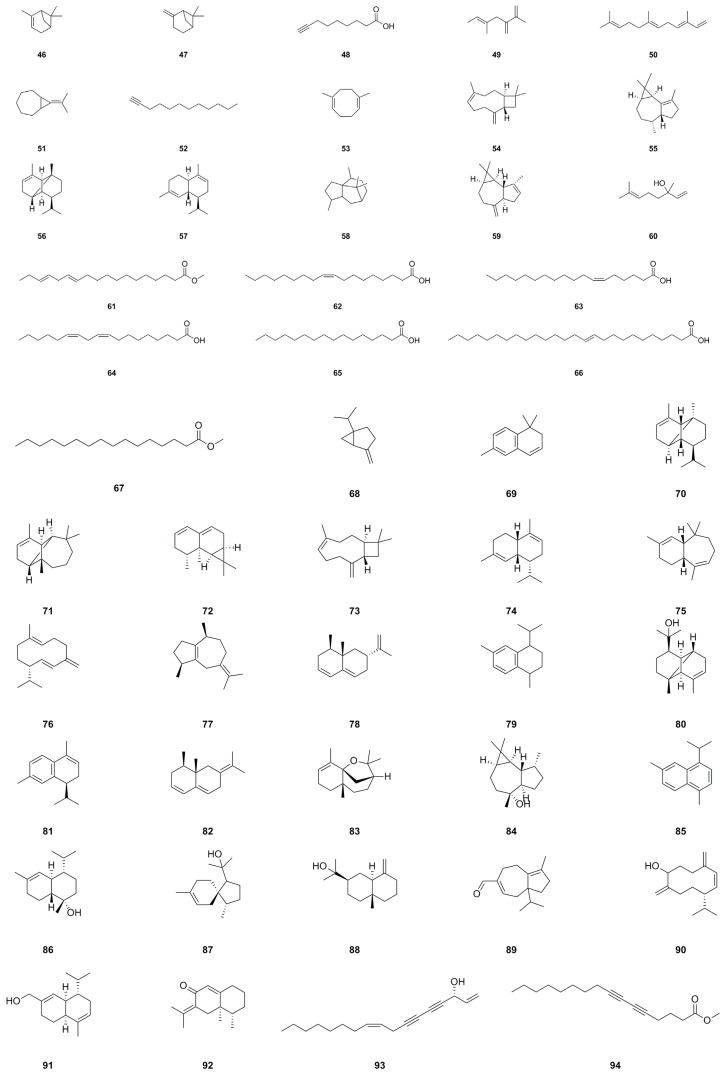
The chemical structures of volatile components from *Aralia continentalis* were accurately depicted using ChemDraw Professional 15.0 software.

To date, forty-nine volatile compounds (**46**–**94**) have been identified and studied from *A. continentalis* (Table 3, Figure 5), offering extensive possibilities for further exploration of their therapeutic and bioactive attributes. Out of these, sixteen compounds were identified in the volatile oil extracted from *A. continentalis* roots, accounting for 92.88% of the total content, with *α*-pinene (**46**) comprising 41.22% [24]. Our previous research identified thirty compounds in the GC-MS analysis of the volatile oil from *A. continentalis* roots, accounting for 98.9% of the total composition [25]. These primary constituents include compound **46** (65.0%) and others such as (+)-α-amorphene (**74**), (–)-nootkatene (**78**), *β*-eudesmol (**88**), and more. This volatile oil displayed modest antioxidant properties, achieving scavenging rates of 22.3% and 16.8% for DPPH and ABTS, respectively. Its IC_50_ value for *α*-glucosidase inhibition was 79.2 μg/mL, which was comparable to the positive control acarbose. However, our findings differ from previous reports on volatile components [25], and this variation can be attributed to various factors: (1) Genetic differences affect plants’ response to stimuli, influencing volatile oil synthesis. (2) Environmental factors like light, temperature, and soil affect plant growth and volatile oil synthesis. (3) Plant compounds vary with developmental stages due to changing biological needs. (4) External stimuli, both biotic and abiotic, trigger defense reactions and alter volatile components [116]. In summary, volatile oil composition differs due to genetics, environment, development, and external stimuli, reflecting plant adaptability and survival strategies.

### 5.4. Phenolics

**Figure 6:**
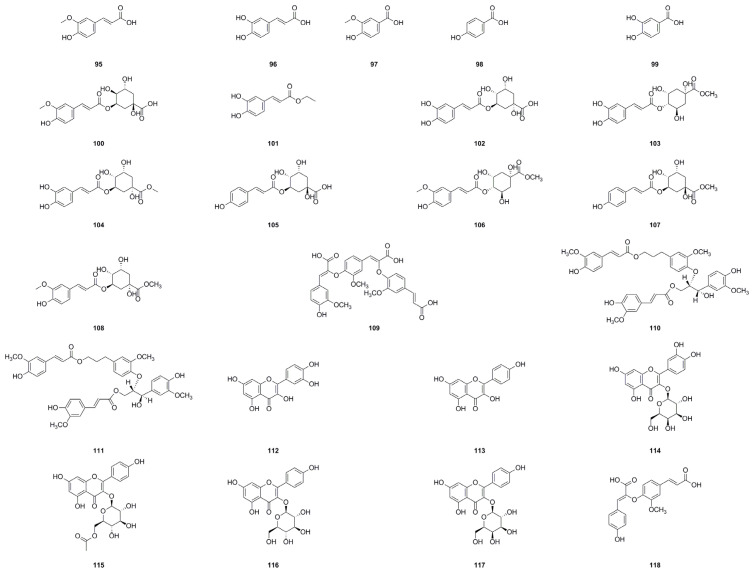
The chemical structures of phenolics from *Aralia continentalis* were accurately depicted using ChemDraw Professional 15.0 software.

In recent years, phenolics have garnered considerable attention owing to their potential role in preventing chronic and degenerative diseases, which are among the leading causes of mortality and disability in developed countries. The intake of phenolics through dietary sources has been linked to several positive health effects [117]. In the roots and leaves of *A. continentalis*, twenty-four phenolics (**95**–**118**) have been effectively extracted and identified, showcasing the plentiful presence of these bioactive compounds in the plant. These findings are detailed in Table 4 and Figure 6.

A recent report has extensively investigated the stabilizing effects of ferulic acid (**95**) and caffeic acid (**96**) on albumin’s heat denaturation [27]. The key findings indicate that, in the context of protein stabilization, the activities displayed by these two phenolic acids are approximately one-eighth of that observed with salicylic acid when comparing 50% inhibition concentrations. In a separate study, researchers isolated ten compounds from the ethyl acetate extract of *A. continentalis* [15]. The results were compelling, revealing that hyperoside (**114**) and quercetin (**112**) possess significant anti-inflammatory activity [15]. Among these compounds, Eo et al. selected a subset for evaluating their effects on the production of nitric oxide (NO) and tumor necrosis factor-alpha (TNF-*α*) in lipopolysaccharide (LPS)-induced RAW264.7 cells [14]. Furthermore, the antioxidant properties of *A. continentalis* leaves were also examined. Specifically, the lipid peroxide production in mouse liver homogenate at 37 °C was measured using thiobarbituric acid and DPPH radical scavenging activity assays. Additionally, chromatographic separation of the homogenate led to the isolation of six flavonoids, among which, quercetin (**112**), hyperoside (**114**), and kaempferol (**113**) exhibited particularly strong antioxidant activities [21].

**Table 4:** Phenolics isolated or identified from *Aralia continentalis*.

No.	Name	Formula	Exact Theoretical M. W.	Source	Characterization Method	Ref.
95.	Ferulic acid	C_10_H_10_O_4_	194.0579	roots	Chemical reaction, TLC, mp, UV, IR, ^1^H NMR	[27]
96.	Caffeic acid	C_9_H_8_O_4_	180.0423	roots	Chemical reaction, TLC, mp, UV, IR, ^1^H NMR	[27]
				roots	ESI-MS, ^1^H NMR, ^13^C NMR	[9]
97.	Vanillic acid	C_8_H_8_O_4_	168.0423	roots	EI-MS, ^1^H NMR, ^13^C NMR	[1]
98.	4-Hydroxybenzoic acid	C_7_H_6_O_3_	138.0317	roots	EI-MS, ^1^H NMR, ^13^C NMR	[1]
99.	Protocatechuic acid	C_7_H_6_O_4_	154.0266	roots	EI-MS, ^1^H NMR, ^13^C NMR	[1]
				roots	ESI-MS, ^1^H NMR, ^13^C NMR	[9]
100.	5-*O*-Feruloyl quinic acid	C_17_H_20_O_9_	368.1107	roots	EI-MS, ^1^H NMR, ^13^C NMR	[1]
101.	Ethyl caffeate	C_11_H_12_O_4_	208.0736	roots	ESI-MS, ^1^H NMR, ^13^C NMR	[9]
102.	Chlorogenic acid	C_16_H_18_O_9_	354.0951	roots	ESI-MS, ^1^H NMR, ^13^C NMR	[9]
103.	Cryptochlorogenic acid methyl ester	C_17_H_20_O_9_	368.1107	roots	ESI-MS, ^1^H NMR, ^13^C NMR	[9]
104.	Chlorogenic acid methyl ester	C_17_H_20_O_9_	368.1107	roots	ESI-MS, ^1^H NMR, ^13^C NMR	[9]
105.	3-*O*-Coumaroylquinic acid	C_16_H_18_O_8_	338.1002	roots	ESI-MS, ^1^H NMR, ^13^C NMR	[9]
106.	4-*O*-Feruloylquinic acid methyl ester	C_18_H_22_O_9_	382.1264	roots	ESI-MS, ^1^H NMR, ^13^C NMR	[9]
107.	3-*O*-Coumaroylquinic acid methyl ester	C_17_H_20_O_8_	352.1158	roots	ESI-MS, ^1^H NMR, ^13^C NMR	[9]
108.	3-*O*-Feruloylquinic acid methyl ester	C_18_H_22_O_9_	382.1264	roots	ESI-MS, ^1^H NMR, ^13^C NMR	[9]
109.	8-*O*-4/8-*O*-4-Dehydrotriferulic acid	C_30_H_26_O_12_	578.1424	roots	ESI-MS, ^1^H NMR, ^13^C NMR	[9]
110.	*erythro*-Carolignan E	C_40_H_42_O_13_	730.2625	roots	ESI-MS, ^1^H NMR, ^13^C NMR	[9]
111.	*threo*-Carolignan E	C_40_H_42_O_13_	730.2625	roots	ESI-MS, ^1^H NMR, ^13^C NMR	[9]
112.	Quercetin	C_15_H_10_O_7_	302.0427	roots	^1^H NMR, ^13^C NMR	[15]
				roots	mp, UV, IR, MS, ^1^H NMR	[28]
				roots	UV, IR, ^1^H NMR, ^13^C NMR	[29]
				leaves	mp, IR, MS, ^1^H NMR, ^13^C NMR	[21]
				roots	UV, IR, ^1^H NMR, ^13^C NMR	[29]
113.	Kaempferol	C_15_H_10_O_6_	286.0477	roots	mp, UV, IR, MS, ^1^H NMR	[28]
				leaves	mp, IR, MS, ^1^H NMR, ^13^C NMR	[21]
114.	Hyperoside	C_21_H_20_O_12_	464.0955	roots	Chemical reaction, ^1^H NMR, ^13^C NMR	[15]
				leaves	mp, IR, MS, ^1^H NMR, ^13^C NMR	[21]
				roots	mp, UV, IR, MS, ^1^H NMR, ^13^C NMR	[28]
115.	6″-*O*-Acetylastragalin	C_23_H_22_O_12_	490.1111	roots	mp, UV, IR, MS, ^1^H NMR, ^13^C NMR	[28]
				leaves	mp, IR, MS, ^1^H NMR, ^13^C NMR	[21]
116.	Astragalin	C_21_H_20_O_11_	448.1006	roots	mp, UV, IR, MS, ^1^H NMR, ^13^C NMR	[28]
				leaves	mp, IR, MS, ^1^H NMR, ^13^C NMR	[21]
117.	Trifolin	C_21_H_20_O_11_	448.1006	roots	mp, UV, IR, MS, ^1^H NMR, ^13^C NMR	[28]
				leaves	mp, IR, MS, ^1^H NMR, ^13^C NMR	[21]
118.	Aralianic acid	C_19_H_16_O_7_	356.0896	roots	UV, IR, ESI-MS, HRESIMS, ^1^H NMR, ^13^C NMR, HMBC, DEPT, HSQC	[9]

UV: Ultraviolet spectrophotometry; mp: Melting point; IR: Infrared spectroscopy; TLC: Thin-layer chromatography; MS: Mass spectrometry; ESI-MS: Electrospray ionization mass spectrometry; HRESIMS: High-resolution electrospray ionization mass spectrometry; ^1^H NMR: Hydrogen-1 nuclear magnetic resonance spectrometry; ^13^C-NMR: Carbon-13 nuclear magnetic resonance spectrometry; HMBC: ^1^H Detected heteronuclear multiple-bond correlation; HSQC: Heteronuclear singular-quantum correlation; DEPT: Distortionless enhancement by polarization transfer.

### 5.5. Vitamins and Trace Elements

**Figure 7:**
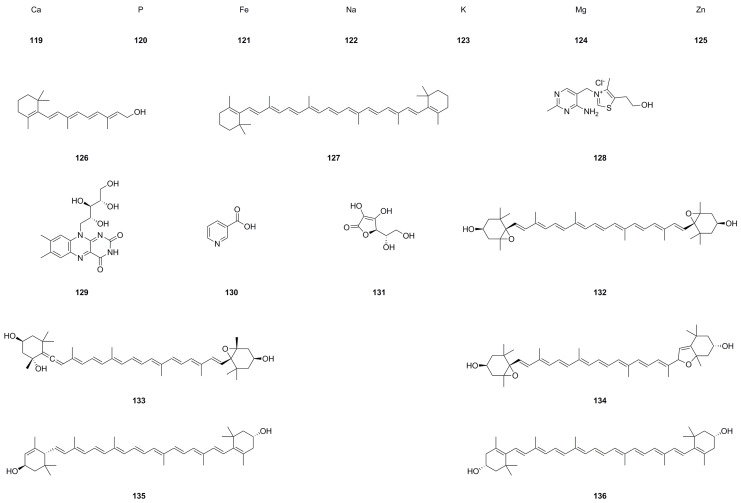
The chemical structures of vitamins and trace elements from *Aralia continentalis* were accurately depicted using ChemDraw Professional 15.0 software.

Eighteen vitamins and trace elements (**119**–**136**) have been effectively extracted and characterized from the roots and leaves of *A. continentalis*, as detailed in Table 5 and Figure 7. A comprehensive report investigated the nutritional and functional components of this plant, revealing its abundance of various nutrients [30]. Generally speaking, the content of water, carbohydrates, crude fiber, and crude protein is relatively high. Among the inorganic components, potassium (**123**) holds the topmost concentration, followed by calcium (**119**), phosphorus (**120**), natrium (**122**), and magnesium (**124**). In terms of vitamins, *β*-carotene (**127**) is found to have the highest content. The crude saponin content in both the roots and leaves is similar, exhibiting strong antioxidant activity. Furthermore, the dietary fiber and chlorophyll content of both the roots and leaves are also significant. These findings indicate that *A. continentalis* possesses potential nutritional value and functional characteristics, making it a promising source for various applications.

### 5.6. Other Compounds

**Figure 8:**
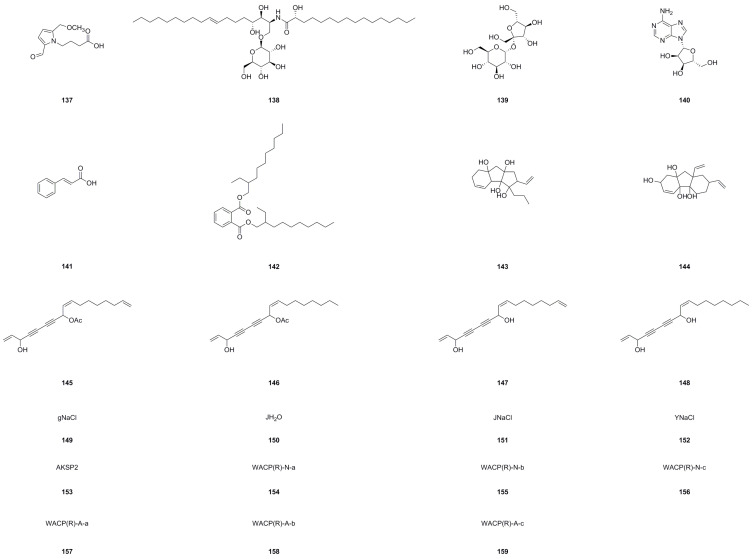
The chemical structures of other compounds from *Aralia continentalis* were accurately depicted using ChemDraw Professional 15.0 software.

In addition to the compound types mentioned earlier, researchers have successfully isolated and identified an additional twenty-three unique compounds (**137**–**159**) from different parts of *A. continentalis*, including its roots, leaves, and stems (Table 6 and Figure 8). These remarkable discoveries emphasize the extraordinary diversity and intricacy of the bioactive compounds harbored within *A. continentalis*, further highlighting its immense potential for exploration and utilization in the fields of pharmacology and medical research.

Eo and colleagues investigated the structure of HY251 (**143**) isolated from *A. continentalis* roots and its inhibitory effects on HeLa cells [32]. Flow cytometry analysis revealed G1 phase arrest in compound **143**-treated HeLa cells, attributed to reduced cyclin D3 levels, increased expression of p21(CIP1) and p27(KIP1), and p53 phosphorylation through ATM upregulation. This led to enhanced hypophosphorylated pRb levels. An additional study exploring the apoptotic mechanisms of compound **143** in prostate cancer LNCaP cells through TUNEL assay and Western blot analysis showed that treatment with 95 μM HY251 for 24 h induced apoptosis. Apoptosis in LNCaP cells was associated with cytochrome C release, caspase activation, and inhibition of androgen receptor and prostate-specific antigen expression [118]. This evidence identified compound **143** as a potential therapeutic agent for both androgen-sensitive and hormone-refractory prostate cancer. Interestingly, Su et al. delved into compound **143**′s apoptotic mechanisms in ovarian cancer PA-1 cells, demonstrating apoptotic induction in cells treated with 60 μM compound **143** for 24 h. This apoptotic process involved caspase-8-dependent Bid cleavage, leading to tBid formation, as well as the activation of caspase-9/-3 and PARP cleavage, with concomitant upregulation and activation of p53 [119]. Overall, these findings suggest the use of compound **143** as a promising chemoterapic option also for ovarian cancer. Another investigation elucidated the structure of HY253 (**144**) from *A. continentalis* roots and its apoptotic effects on HeLa cells [33]. Treatment with compound **144** induced apoptosis in HeLa cells through cytochrome c release, the upregulation of pro-apoptotic Bcl-2 proteins, and the activation of various caspases and PARP cleavage. Additionally, compound **144** was found to induce G1 phase arrest in human lung cancer A_549_ cells [120]. The anti-cancer effect of compound **144** was associated with the altered expression of cell cycle proteins and apoptosis induction through cytochrome C release, caspase activation, and p53 induction. Subsequent research was conducted focusing on compound **144′**s molecular mechanisms in cell cycle regulation and apoptosis in human liver cancer HepG2 cells. The study revealed G1 phase arrest, the upregulation of CDK inhibitors, and apoptosis induction marked by cytochrome c release, the downregulation of Bcl-2, caspase activation, and PARP cleavage upon HY253 treatment [121]. Thanks to this evidence, HY253 emerged as a promising chemotherapeutic candidate for liver cancer via p53 activation. This indication, together with the experimental evidence reported so far, highlights the potential of using HY251 and HY253 as effective chemotherapic agents across multiple cancer types.

The initial research on polysaccharides revealed that the root polysaccharides of *A. continentalis* consist of rhamnose, arabinose, mannose, glucose, and uronic acid [122]. This led to the isolation of four distinct polysaccharides from *A. continentalis* (Table 6, Figure 8), namely, gNaCl (**149**), JH_2_O (**150**), JNaCl (**151**), and YNaCl (**152**), each characterized by *β*-glycoside bonds and sugar-specific absorption peaks but lacking sulfate and acetyl groups. Specifically, gNaCl is comprised of *D*-galactouronic acid, *D*-fructose, and *D*-glucose; JH_2_O contains *D*-mannose and *D*-glucose; JNaCl is composed of *D*-glucose and *D*-galactose; and YNaCl consists of *D*-galactouronic acid and *D*-sorbose [123]. Moreover, a separate study focused on soluble polysaccharides extracted from *A. continentalis* roots identified the polysaccharide AKSP2 (**153**). HPLC analysis confirmed that AKSP2 is composed of xylose, fructose, mannose, and glucose, while infrared spectroscopy indicated it as a furan glycoside with *β*-type glycosidic bonds, including acetyl groups [124]. The administration of AKSP2 at 200 mg/kg significantly elevated superoxide dismutase (SOD) and catalase (CAT) levels in mouse liver and serum, indicating enhanced antioxidant capacity and decreased malondialdehyde (MDA) values, suggesting its antioxidant and potential anti-aging effects [124]. Additionally, the isolation of polysaccharide WACP (R) from *A. continentalis* roots using ion exchange chromatography led to the identification of neutral sugar WACP (R)-N and acidic sugar WACP (R)-A [125]. Purification revealed that WACP (R)-N is further divided into three components, with WACP (R)-N-a (**154**) showing consistent molecular weight. On the other hand, WACP (R)-A is subdivided into three components, with both WACP (R)-A-a (**157**) and WACP (R)-A-c (**159**) demonstrating uniform molecular weights. Notably, WACP (R)-A-c was identified as a polygalacturonic acid-type pectin. In vitro antioxidant assays demonstrated that WACP (R) and WACP (R)-A exhibit antioxidant properties, with WACP (R)-A-c displaying the highest efficacy in scavenging DPPH free radicals [126], thereby establishing it as the primary antioxidant constituent. These findings support the exploration of natural polysaccharide-based antioxidants and advocate for the utilization of *A. continentalis*.

**Table 6:** Other compounds isolated or identified from *Aralia continentalis*.

No.	Name	Formula	Exact Theoretical M. W.	Source	Characterization Method	Ref.
137.	4-[Formy-5-(methoxymethyl)-1H-pyrrol-1-yl] butanoic acid	C_11_H_15_NO_4_	225.1001	roots	FAB-MS, HR FAB-MS, ^1^H NMR, ^13^C NMR	[1]
138.	Aralia cerebroside	C_40_H_77_NO_10_	731.5547	roots	mp, EI-MS, ^1^H NMR, ^13^C NMR	[1]
139.	Sucrose	C_12_H_22_O_11_	342.1162	roots	Chemical reaction, ^1^H NMR, ^13^C NMR	[15]
140.	Adenosine	C_10_H_13_N_5_O_4_	267.0968	leaves	mp, IR, MS, ^1^H NMR, ^13^C NMR	[21]
141.	Cinnamic acid	C_9_H_8_O_2_	148.0524	roots	ESI-MS, ^1^H NMR, ^13^C NMR	[9]
142.	*ortho*-Phthalic acid *bis*-(2-ethyldecyl)-ester	C_32_H_54_O_4_	502.4022	roots	Chemical reaction, ^1^H NMR, ^13^C NMR	[16]
143.	HY251	C_17_H_26_O_4_	294.1831	roots	^1^H NMR, ^13^C NMR, ^1^H–^1^H COSY, HMQC, HMBC	[32]
144.	HY253	C_17_H_24_O_4_	292.1675	roots	^1^H NMR, ^13^C NMR, ^1^H–^1^H COSY, HMQC, HMBC	[33]
145.	Dehydrofalcarindiol-8-acetate	C_19_H_24_O_3_	300.1725	roots	ESI-MS, ^1^H NMR, ^13^C NMR	[9]
146.	Falcarindiol-8-acetate	C_19_H_26_O_3_	302.1882	roots	ESI-MS, ^1^H NMR, ^13^C NMR	[9]
147.	Dehydrofalcarindiol	C_17_H_22_O_2_	258.1620	roots	ESI-MS, ^1^H NMR, ^13^C NMR	[9]
148.	Falcarindiol	C_17_H_24_O_2_	260.1776	roots	ESI-MS, ^1^H NMR, ^13^C NMR	[9]
149.	gNaCl	—	—	roots	Paper chromatography, IR, HPLC	[123]
150.	JH_2_O	—	—	stems	Paper chromatography, IR, HPLC	[123]
151.	JNaCl	—	—	stems	Paper chromatography, IR, HPLC	[123]
152.	YNaCl	—	—	leaves	Paper chromatography, IR, HPLC	[123]
153.	AKSP2	—	—	roots	IR, HPLC	[124]
154.	WACP(R)-N-a	—	172.8 KDa	roots	IR, HPLC, scanning electron microscopy analysis	[125,126]
155.	WACP(R)-N-b	—	7.0 KDa	roots	IR, HPLC, scanning electron microscopy analysis	[125,126]
156.	WACP(R)-N-c	—	<1.2 KDa	roots	IR, HPLC, scanning electron microscopy analysis	[125,126]
157.	WACP(R)-A-a	—	171.1 KDa	roots	IR, HPLC, scanning electron microscopy analysis	[125,126]
158.	WACP(R)-A-b	—	60.8 KDa	roots	IR, HPLC, scanning electron microscopy analysis	[125,126]
159.	WACP(R)-A-c	—	27.3 KDa	roots	IR, HPLC, scanning electron microscopy analysis	[125,126]

mp: Melting point; IR: Infrared spectroscopy; HPLC: High-performance liquid chromatography; MS: Mass spectrometry; EI-MS: Electron impact mass spectrometry; FAB-MS: Fast atom bombardment–mass spectrometry; HR FAB-MS: High-resolution fast atom bombardment–mass spectrometry; ESI-MS: Electrospray ionization mass spectrometry; ^1^H NMR: Hydrogen-1 nuclear magnetic resonance spectrometry; ^13^C-NMR: Carbon-13 nuclear magnetic resonance spectrometry; COSY: Correlation spectroscopy; HMBC: ^1^H Detected heteronuclear multiple-bond correlation; HMQC: ^1^H Detected heteronuclear multiple-quantum coherence.

## 6. Pharmacological Effects

Modern pharmacological studies have established that *A. continentalis*, as a medicinal plant, possesses diverse pharmacological effects. These include anti-inflammatory and analgesic properties, antioxidant capabilities, antimicrobial activity, insecticidal qualities, hepatoprotective effects, anti-diabetic potential, cytotoxic properties, as well as other pharmacological benefits.

### 6.1. Anti-Inflammatory and Analgesic Effects

In a study by Li et al., the researcher investigated the antioxidant and anti-inflammatory properties of ethanol extracts derived from *A. continentalis* using an in vitro setting. The analyzed extracts were found to contain 12.88 mg/g of polyphenols and 4.54 mg/g of flavonoids, demonstrating their strong radical-scavenging abilities and anti-inflammatory properties related to the inhibition of NO production [34]. A 50% ethanol extract of *A. continentalis* showed significant inhibitory effects in various circumstances, including carrageenan-induced inflammation, egg white-induced responses, scalding effects, formaldehyde-induced foot swelling, exudate production in croton oil gas cysts, and granulation tissue proliferation [35]. *A. continentalis* extract also notably suppressed adjuvant arthritis and allergic inflammation. Additionally, apart from impacting the Arthur reaction, the extract exhibited inhibitory effects on both humoral and cellular immunity, significant suppression of inflammatory mediators, and related consequential effects during the later stages of allergic reactions [35]. Moreover, the 50% ethanol extract of *A. continentalis* displayed a range of pharmacological activities, such as the reduction of mouse activity, the protraction of pentobarbital sodium-induced sleep time and its sub-threshold hypnotic dose, significant anti-convulsive effects, and pain alleviation induced by chemical, thermal, and electrical stimuli. It was also observed to lower body temperature in normal rats and significantly suppress fever induced by yeast suspension in rats. Therefore, this extract emerges as a potent agent with considerable antipyretic, analgesic, sedative, and anticonvulsant properties [36]. Indeed, the volatile oil extracted from *A. continentalis* notably decreased spontaneous activity in mice, extended the sleep duration induced by pentobarbital sodium and its sub-threshold dose, and exhibited a substantial antagonistic effect against convulsions triggered by strychnine, pentylenetetrazol, and caffeine [37]. Investigating the inhibitory impacts of *A. continentalis* root on histamine release from mast cells and NO production by macrophages, Wang and team noted a 32.7% inhibition rate for histamine release at a concentration of 100 μg/mL. Simultaneously, the inhibition rate for NO production at this same concentration level was recorded as 48.1% [38]. Additionally, the *A. continentalis* extract stimulated murine macrophages (RAW264.7), inducing various immune responses such as NO production, cytokine secretion, phagocytosis, and the generation of reactive oxygen species [39]. Immunoblot analysis hinted at the involvement of NF-*κ*B and MAPK signaling pathways in mediating these immunostimulatory effects, suggesting that the *A. continentalis* extract holds promise in augmenting macrophage function and could potentially serve as a therapeutic option for immune-related disorders [39].

Comparing the inhibitory effects of *A. continentalis* root on inflammatory mediators and swelling from chemically induced ear edema, Cheon and coauthors revealed compelling findings. The results indicated that *A. continentalis* root effectively inhibited the release of NO, prostaglandin E2 (PGE2), interleukin-1*β*, and TNF-*α* in macrophages. Additionally, *A. continentalis* root demonstrated efficacy in reducing edema induced by 12-*O*-tetradecanoylphorbol-13-acetate in mouse ears [40]. Overall, the study underscored the potent anti-inflammatory activities exhibited by *A. continentalis* root. In a separate investigation, the 80% ethanol extract of *A. continentalis* root was analyzed for its impact on complete Freund’s adjuvant (CFA)-induced arthritis in rats [41]. Remarkably, *A. continentalis* root significantly suppressed nociceptive behaviors and Fos expression in the spinal cord, indicating its potential to alleviate arthritis symptoms in rats and, potentially, humans. Furthermore, an additional study elucidated the anti-arthritic activity of a combination of *Ostericum koreanum* (OS) and *A. continentalis* in vivo [42]. Mice immunized with bovine type II collagen were administered daily with an OS plus *A. continentalis* extract mixture for 7 weeks. The oral administration of this combination significantly impeded the progression of collagen-induced arthritis (CIA), akin to the effects of methotrexate. Histological evaluation revealed reduced cartilage destruction and pannus formation. Moreover, the OS plus *A. continentalis* mixture exhibited inhibitory effects on TNF-*α* and IL-6 production in serum, decreased specific immune cell populations in paw joints, and modulated cytokine levels in splenocyte cultures [42]. These outcomes underscored the potent anti-inflammatory and immunomodulatory effects of OS plus *A. continentalis* mixture in suppressing the progression of CIA. Developing IL-6 receptor antagonists or interfering with STAT3 activation appears, therefore, an effective method for treating IL-6-related diseases, as supported by Oh et al. In the study, the author isolated continentalic acid (**1**) from *A. continentalis* and studied its impact on the IL-6 reaction, demonstrating that compound **1** can inhibit the IL-6-induced phosphorylation of STAT3 and ERK1/2 in Hep3B cells [43]. Therefore, compound **1** exhibits strong IL-6 signaling and IL-6 production inhibitory activity and is expected to become a candidate drug for the treatment of inflammatory diseases.

In a related study, the ethanol extract of *A. continentalis* roots was scrutinized for its impact on NO production in RAW264.7 cells and inflammatory symptoms in mice [44]. The results showcased that *A. continentalis* roots not only suppressed NO production in LPS-activated cells but also exhibited remedial effects against gastritis and hepatitis in mice. The anti-inflammatory effect of *A. continentalis* roots is believed to result from inhibiting the NF-*κ*B pathway, which involves Syk and Src at the transcriptional level. This indicates the possibility of using *A. continentalis* roots as a herbal remedy targeting Syk- and Src-mediated anti-inflammatory processes. However, additional preclinical research is needed to confirm these results. The ethanol extract derived from *A. continentalis* was also examined for its anti-inflammatory and anti-nociceptive properties. In fibroblast-like synoviocytes stimulated by IL-1*β* in rheumatoid arthritis patients, *A. continentalis* extract promoted a reduction in inflammatory mediators such as TNF-*α*, IL-6, IL-8, MMP-1, and MMP-13 [45]. Furthermore, it exhibited inhibition of NF-*κ*B migration into the nucleus by suppressing the MAPK pathway. In another experiment involving collagen-induced polyarthritis mice, the *A. continentalis* extract was found to alleviate arthritic symptoms and behaviors. However, it did not show significant effects on carrageenan-induced hyperalgesia or thermal nociception in rats [45]. In summary, the ethanol extract of *A. continentalis* displays notable anti-inflammatory and anti-arthritic effects in rodent arthritis models and in vitro studies. Studies have shown that the total saponins of *A. continentalis* can reduce NLRP3, IL-1*β*, and IL-18 levels in rat myocardial tissue, offering protection to myocardial tissue, though the specific mechanism remains unclear [48]. Further investigations, aimed at developing a rat heart ischemia–reperfusion injury model by intervening in the PI3K/AKT signaling pathway revealed that *A. continentalis* saponins reduced the expression of NLRP3-related proteins and genes [49]. Interestingly, this effect was hindered when total saponins were co-administrated with the PI3K inhibitor LY29004 [49]. This suggests that the *A. continentalis* total saponins influence NLRP3 inflammasomes by modulating the PI3K/AKT signaling pathway to combat myocardial ischemia–reperfusion injury. Post-traumatic stress disorder (PTSD) is a condition triggered by traumatic stress, leading to prolonged stress responses, memory impairment, and inflammation in the hippocampus. Conventional antidepressants for treating PTSD have limited efficacy. The potential impact of *A. continentalis* on cognitive memory and PTSD mechanisms remains uncertain. To delve into this, an examination was conducted on the effect of *A. continentalis* on spatial cognitive impairment in a rat model of PTSD induced by single prolonged stress. Treatment with *A. continentalis* at 100 mg/kg reversed cognitive deficits, boosted brain-derived neurotrophic factor levels in the hippocampus, and mitigated inflammation triggered by single prolonged stress [50]. These findings suggest that *A. continentalis* holds promise as an intervention for PTSD and warrants further exploration.

Jung and colleagues attempted to enhance the anti-inflammatory effect of fermented *A. continentalis* root (fACR) by utilizing selected *Lactobacillus* strains for fermentation, resulting in increased levels of polyphenols and amino acids in fACR compared to the aqueous extract of *A. continentalis* root [51]. The anti-inflammatory activity of fACR was further assessed by measuring cytokines and MMP-9 activity associated with arthritis, revealing that *Lactobacillus* fermentation augments the biological activities of *A. continentalis* root and enhances its inflammation-inhibiting effects. These findings established the groundwork for investigating the preventative potential of fACR against arthritis. Woo et al. examined extracts of *A. continentalis*, with and without *Lactobacillus plantarum* fermentation, to evaluate their anti-inflammatory effects. Various extracts were prepared using water, ethanol, hexane, ethyl acetate, and butanol and tested on RAW264.7 macrophages induced by LPS for toxicity and anti-inflammatory activity [52]. These extracts significantly inhibited NO production, COX-2 and iNOS mRNA expression, and cytokine production. Notably, extracts fermented with *Lactobacillus plantarum* appeared promising for controlling the expression of inflammatory cytokine genes, whereas, post-fermentation, water, ethanol, and butanol extracts showed potential as functional natural materials with anti-inflammatory properties [52].

*Ent*-kaur-16-en-19-oic acid (**14**) was isolated from *A. continentalis* as an active compound, demonstrating albumin-stabilizing activity with an IC_50_ of 0.026 mg/3 mL. In a carrageenin-induced edema test, compound **14** showcased anti-inflammatory activity, with subcutaneous administration slightly less effective but oral administration three times more effective than phenylbutazone. These findings contrast with the outcomes of another active component, *ent*-pimara-8(14),15-dien-19-oic acid (**19**), concerning administration routes [53]. In a study by Lim et al., the anti-inflammatory effects of five constituents from *A. continentalis* roots were studied. 7-oxo-ent-pimara-8(14),15-diene-19-oic acid (**6**) and kaurenoic acid (**2**) demonstrated moderate inhibition of NO production, whereas compound **6** weakly suppressed PGE2 production [8]. Through Western blot and electrophoretic assays, it was found that compound **6** mildly inhibited COX-2 and iNOS expression and NF-*κ*B activation. Furthermore, in vivo experiments revealed that continentalic acid (**1**) and compound **2** significantly reduced carrageenan-induced paw edema in mice. Overall, certain constituents of *A. continentalis*, particularly compounds **1**, **2**, and **6**, exhibit anti-inflammatory activity, contributing to the overall therapeutic effects of its roots.

In another study, the objective was to identify the active compound in *A. continentalis* for its pharmacological effects [54]. IL-1*β*-stimulated human chondrocytes and osteoarthritic rats were treated with an *A. continentalis* extract or its components such as continentalic acid (**1**) and kaurenoic acid (**2**). The extract suppressed the IL-1*β*-induced production of several inflammatory markers and kinases, excluding MMP-3. Compound **1** displayed similar anti-arthritic activity to the extract, while a higher concentration was required for compound **2**. Both the extract and compound **1** proved effective in osteoarthritic rats, suggesting the promising anti-arthritic activities of *A. continentalis* extract, with compound **1** potentially being the key contributor to its efficacy [54]. Studying the anti-inflammatory properties of compounds derived from the roots of *A. continentalis* in RAW264.7 cells, Hong and colleagues identified a novel compound, 18-nor-*ent*-pimara-9(11),15-diene-4*β*-ol, which exhibited significant anti-inflammatory activity. This compound effectively blocked the production of NO and inhibited the expression of iNOS, COX-2, TNF-*α*, and IL-1*β* in LPS-stimulated cells. Furthermore, it suppressed the activation of NF-*κ*B and attenuated the phosphorylation of p38 and ERK1/2 [14]. These findings indicate that 18-nor-*ent*-pimara-9(11),15-diene-4*β*-ol possesses considerable potential for the development of anti-inflammatory drugs.

The analgesic effects of kaurenoic acid (**2**) were investigated across various pain models and mechanisms in mice [55,56]. Compound **2** demonstrated a reduction in inflammatory pain induced by acetic acid, phenyl-*p*-benzoquinone, CFA, and formalin, as well as a decrease in acute and chronic inflammatory hyperalgesia. Additionally, it was found to suppress hyperalgesic cytokines and activate a signaling pathway for pain relief, highlighting its potential as an analgesic agent [55]. Building upon this research, a study was conducted to examine the in vivo effects of compound **2** on LPS-induced inflammation and pain in mice. The results indicated that compound **2** significantly alleviated both mechanical and thermal hyperalgesia, reduced myeloperoxidase activity, and modulated the redox status in the paws of mice. Furthermore, compound **2** inhibited leukocyte migration, cytokine production, oxidative stress, and NF-*κ*B activation during LPS peritonitis, while also enhancing the anti-inflammatory cytokine IL-10 [56]. These findings suggest that compound **2** could serve as a potential treatment for excessive inflammation and pain associated with Gram-negative bacterial infections. Moreover, a study by Kim et al. explored the role of compound **2** in activating Nrf2, a key anti-inflammatory factor, and its potential therapeutic application against neutrophilic lung inflammation, such as acute lung injury. The findings revealed that compound **2** administered to the lungs improved lung structure, reduced neutrophil infiltration and myeloperoxidase activity, and suppressed pro-inflammatory cytokines. Notably, Nrf2 activation by compound **2** was found to be crucial for its anti-inflammatory effects, as evidenced by the limited efficacy in Nrf2 knockout mice. In a sepsis model, compound **2** significantly reduced mortality rates, highlighting its therapeutic potential against inflammatory lung diseases [57]. Further investigations focused on the anti-inflammatory effects of compound **2** by examining its impact on Nrf2 activation. Lyu and colleagues revealed that kaurenoic acid activated Nrf2, resulting in the expression of Nrf2-dependent genes like GCLC and HO-1, while showing no effect on NF-*κ*B activation or the expression of pro-inflammatory mediators. These results suggest that the benefits of *A. continentalis* root extract are partially mediated by Nrf2 activation [13].

Lastly, a study hypothesized that kaurenoic acid suppresses inflammation by activating TGF-*β* signaling [58]. The research demonstrated that compound **2** induced Smad2/3 phosphorylation and transcriptional activity, leading to increased *α*-SMA expression and the activation of TGF-*β* signaling in mouse lungs. This mechanism provides a new perspective on the anti-inflammatory function of compound **2**. An additional study investigated the anti-inflammatory effects of compound **2** on LPS-induced RAW264.7 macrophages and carrageenan-induced paw edema in mice [59]. The results showed that compound **2** inhibited NO production, PGE2 release, COX-2 and iNOS expression in macrophages, and NF-*κ*B activation, while also reducing paw swelling in the edema model through the inhibition of iNOS and COX-2 expression, further highlighting its anti-inflammatory properties.

### 6.2. Antioxidant Activity

Research findings have underscored the strong antioxidant properties exhibited by *A. continentalis* root. In different experimental investigations, *A. continentalis* extract demonstrated remarkable DPPH scavenging activity, the inhibition of linoleic acid peroxidation, and a higher phenolic content compared to *Angelica pubescens* root [60]. In a mouse model with induced oxidative stress, *A. continentalis* root significantly elevated glutathione (GSH) and GSH peroxidase activity while reducing SOD activity, suggesting its potential as a potent antioxidant agent [60]. Further analysis delved into the biological activity of methanol extracts from cultivated and adventitious roots of *A. continentalis*. The methanol extract from adventitious roots exhibited the highest antioxidant activity, with IC_50_ values of 175.6 and 279.7 μg/mL for DPPH and ABTS, respectively. Additionally, this extract showed antibacterial activity against various strains, including *S. aureus*, *S. epidermidis*, *B. subtilis*, and *S. enterica*, supporting the notion that adventitious roots of *A. continentalis* hold promise as natural antioxidant and antibacterial agents [61]. Moreover, the 80% ethanol extracts of *A. continentalis* displayed moderate scavenging abilities for peroxyl radicals, hydroxyl radicals, and peroxynitrite [62]. Conversely, the antioxidant activity of *A. continentalis* aqueous extract was relatively weak, with IC_50_ values for DPPH and ABTS scavenging greater than 200 μg/mL [63]. The aqueous extract of *A. continentalis* root has a certain total phenolic content, DPPH and ABTS scavenging ability, and weak xanthine oxidase inhibitory activity [64]. After freeze-drying, the stem and bud of *A. continentalis* (inhibition rate 79.3%) showed very strong antioxidant activity. In fact, Fe^2+^-induced TBARS formation was almost completely suppressed in brain homogenates. When the stem and bud showing strong antioxidant activity were treated with heat, antioxidant activity increased in the brain tissues to 83.6% [65].

An additional investigation demonstrated that upon reaching a total flavonoid concentration of 0.15 mg/mL in *A. continentalis* roots, the capacity to scavenge DPPH, hydroxyl radicals, and superoxide anion radicals showed remarkable enhancement, exceeding that of vitamin C. The free radical scavenging rates were impressive, with rates of 96.3%, 70.1%, and 44.1%, respectively [66]. Exploring the saponins present in *A. continentalis* leaves and tender buds, it was found that the 30% and 50% ethanol-eluted saponins from leaves had the capacity to scavenge superoxide anion free radicals, with IC_50_ values of 382.8 μg/mL and 344.9 μg/mL, while displaying antibacterial activity against *Escherichia coli* and *Staphylococcus aureus* [67]. Similarly, the 50% and 70% ethanol-eluted saponins from tender buds exhibited a scavenging ability against superoxide anion radicals, with IC_50_ values of 356.44 μg/mL and 501.85 μg/mL, along with antibacterial activity against the same bacterial strains [68]. In a study investigating the antioxidant activities and extraction yield increase of gamma-irradiated *A. continentalis* for health and beauty food resources, gamma irradiation significantly enhanced photoluminescence and thermoluminescence, reflective of irradiation effects. Electron spin resonance analysis confirmed the effects of irradiation. The highest phenolic content was observed in aqueous extracts of non-irradiated samples and 50% ethanol extracts of irradiated samples. Notably, all extracts exhibited substantial antioxidant activities, including DPPH scavenging, ABTS radical cation inhibition, and antioxidant protection [69]. The purpose of the study was to create composite packaging films that could better preserve freshness by incorporating *A. continentalis* root extract (ARE) and chitosan (CH) into their composition. Films with varying ARE concentrations (0.05%, 0.10%, and 0.15% w/v) exhibited high antioxidant capacity, with increased DPPH and ABTS free radical scavenging rates. Surface microstructure changes indicated good compatibility between CH and ARE. In summary, ARE can be combined with CH to form films that effectively enhance the performance of composite packaging [70]. Another study on ARE’s effects on rat chondrocyte viability and cartilage metabolism mRNA under oxidative stress showed that AREs from ethanol and hot water were non-toxic. Under oxidative stress, AREs improved chondrocyte viability, with the 80% ethanol extract being the most effective. It upregulated cartilage synthesis genes and downregulated catabolism genes. The 80% ethanol extract best preserves chondrocyte viability and promotes cartilage synthesis [71].

Different authors conducted a series of studies focusing on the beneficial effects of polysaccharides from *A. continentalis* roots in various animal models [72,125,126]. In a mouse model, researchers investigated the impact of different dosages (100 and 200 mg/kg) of *A. continentalis* polysaccharide on antioxidant function. The results demonstrated a significant decrease in MDA levels in both the kidneys and serum, coupled with increased activities of SOD and CAT. These outcomes highlight the potent antioxidant properties of *A. continentalis* polysaccharides in mitigating oxidative stress in model mice [72]. Furthermore, another study aimed to evaluate the anti-fatigue properties of *A. continentalis* extract in rats subjected to forced swimming. Rats pre-treated with the extract exhibited prolonged swimming duration until exhaustion, indicating enhanced endurance. The extract contributed to maintaining blood homeostasis, reducing lactate accumulation during fatigue, and mitigating muscle injury and apoptosis. The observed anti-fatigue effect of *A. continentalis* extract was attributed to its ability to suppress oxidative stress [73]. The findings suggest that *A. continentalis* extract could serve as a promising therapeutic intervention to combat exercise-induced fatigue. In a separate investigation, the anti-thrombotic potential of *A. continentalis* roots was explored. The study revealed that the methanol extract and its fractions exhibited anti-platelet and antioxidative activities. Among the fractions, the ethyl acetate fraction displayed the most potent inhibition of platelet aggregation and demonstrated radical-scavenging properties. Additionally, this fraction effectively inhibited thrombin-stimulated platelet adhesion, indicating its potential to prevent platelet hyperactivation and serve as a resource for anti-thrombotic therapies [74]. Lastly, the protective effects of continentalic acid (**1**) against nephrotoxicity induced by LPS and *Escherichia coli* were examined. In a study by Khan et al., the administration of compound **1** significantly improved behavioral parameters, renal function, and hematological indices in injured kidneys. Furthermore, it enhanced the activity of antioxidant enzymes while simultaneously reducing oxidative stress markers. The compound effectively mitigated neutrophil infiltration, NO production, and histological damage, thereby protecting the kidneys from inflammatory damage. Additionally, compound **1** demonstrated the ability to prevent DNA damage and modulate the expression of Nrf2 and iNOS, indicating its potential therapeutic value against nephrotoxic insults [75].

### 6.3. Antimicrobial Activity

Dental plaque, which is a biofilm composed of microorganisms that adhere to the tooth surface, significantly influences the onset of cavities and periodontal diseases. Among the microorganisms typically present in dental plaque, *Streptococcus mutans* (*S. mutans*) is notably significant in causing dental problems including cavities and periodontitis. Without proper treatment, these conditions can advance to more serious stages such as gingivitis. Although various chemical agents have been researched for their antimicrobial properties in the mouth, several are linked to side effects that restrict their prolonged usage. Research has highlighted the potential antimicrobial properties of methanol extracts from *A. continentalis* in combating oral pathogens. Choi et al. showed that these extracts exhibit a significant inhibition rate of 89% against *S. mutans* and 11% against *Candida albicans*. This reinforces the notion that *A. continentalis* could serve as an effective agent in preventing dental diseases [76]. Further investigations have delved into the specific mechanisms underlying the antimicrobial effects of *A. continentalis*. For example, studies have demonstrated that the leaf extract of *A. continentalis* displays a clear zone of 9.0 mm, with a minimum inhibitory concentration (MIC) of 100 μg/mL against *S. mutans* [77]. Concentration-dependent inhibitory effects have been observed on various aspects of *S. mutans’* physiology, including growth, acid production, adhesion, and glucan synthesis. Moreover, compounds like acanthoic acid (**3**), continentalic acid (**1**), and kaurenoic acid (**2**) found in *A. continentalis* have been shown to impact the physiology of *S. mutans*, leading to growth defects and alterations in cell membrane composition. RNA-seq analysis has revealed how these diterpenoids modulate gene expression related to essential cellular processes in *S. mutans*, offering new insights into combating dental cavities [78]. The methanol extract of *A. continentalis* has shown remarkable effectiveness in inhibiting the growth of *S. mutans* and *S. sanguinis*, although it does not exhibit the same inhibitory effect on *E. coli*. Through downregulating the gtfD gene and in synergy with compound **2**, the extract has been found to reduce the growth rate of *S. mutans*, impede its biofilm formation, and suppress the expression of virulence-related genes [79]. Moreover, three potent antibacterial compounds present in *A. continentalis*—compounds **1**-**3** have been shown to disrupt the physiology of *S. mutans*, leading to growth abnormalities and changes in cell membrane composition. RNA-seq analysis has revealed how these diterpenoids influence gene expression associated with crucial cellular functions in *S. mutans*, offering novel avenues for combating infections caused by this bacterium, a primary driver of dental cavities [2].

Researchers specifically identified compound **2** from *A. continentalis* and investigated its potential as an anticariogenic agent. Notably, compound **2** displayed significant inhibitory effects on various aspects of *S. mutans’* pathogenicity, including growth, acid production, adhesion, and biofilm formation [80]. Real-time PCR analysis further confirmed a decrease in the expression of virulence genes in *S. mutans* following treatment with compound **2**. These findings suggest that compound **2** holds promise as a valuable agent in the prevention of dental caries. In a separate study, *A. continentalis* was evaluated for its ability to inhibit cariogenic *S. mutans*. The research encompassed assessments of growth rates, acid production, glucan synthesis, adhesion, and biofilm formation. The results indicated a concentration-dependent inhibitory effect on growth and acid production with the ethanol extract of *A. continentalis*. Furthermore, the extract was shown to reduce glucan synthesis, adherence to hydroxyapatite, and the formation of biofilms [81]. These outcomes reinforce the traditional use of *A. continentalis* in dental treatments and suggest its potential to counteract the cariogenic properties associated with *S. mutans*.

In an ongoing quest to discover compounds with potent antibacterial activity against methicillin-resistant *Staphylococcus aureus* (MRSA), researchers identified continentalic acid in a chloroform extract of *A. continentalis* roots. This compound showed significant efficacy against both standard methicillin-susceptible *Staphylococcus aureus* (MSSA) and clinical MRSA isolates. Testing revealed that continentalic acid displayed MICs ranging from approximately 8 to 16 mg/mL against various strains of *S. aureus*, including both MSSA and MRSA [82]. These findings strongly indicate the potential of continentalic acid (**1**) as a promising adjunct in combating antibiotic-resistant bacteria. The scourge of rice sheath blight, caused by the fungus *Rhizoctonia solani*, presents significant global challenges and incurs substantial antifungal control expenses. Research has shown that *A. continentalis* root extract possesses potent antifungal properties against this destructive fungus, believed to be attributed to its *ent*-pimara-8(14),15-diene-19-oic acid content. Field trials have demonstrated that the application of this extract (at concentrations of 125 mg/L or higher) on rice plants can reduce sheath blight damage to less than 16%, establishing it as a viable control agent [83]. Moreover, the methanol extract of the entire plant of *A. continentalis* has exhibited promising inhibitory effects (14 mm) against *Propionibacterium acnes*, showcasing its potential in combating acne [84]. Another report used the paper diffusion method to study the effect of methanol extract from *A. continentalis* fruits on the impact of *E. coli*, *S. typhimurium*, *S. aureus*, and *L. monocytogenes*. It was found that the methanol extract had an effective impact on *S. aureus*, with a clear zone on the plate of 7.0 mm [85].

### 6.4. Insecticidal Activity

In terms of insecticidal properties, methanol extract from *A. continentalis* roots displayed moderate nematicidal activity, resulting in a mortality rate of 22% at a concentration of 1000 μg/mL against *Bursaphelenchus xylophilus* [86]. *Leishmania amazonensis* infection presents grave health risks to humans, with current treatment options being both toxic and ineffective. A study delved into the antiprotozoal impact and mechanisms of kaurenoic acid against *Leishmania amazonensis*. Kaurenoic acid (**2**) was found to directly eliminate promastigotes, reduce amastigote numbers in infected macrophages, restore NO production, disrupt the parasite’s evasion tactics, and induce IL-1*β* production and NLRP12 expression. These findings underscore the leishmanicidal potential of compound **2** against *Leishmania amazonensis* within macrophages, operating through an NLRP12/IL-1*β*/cNOS/NO mechanism [87]. Understanding the pathways activated by natural compounds is vital for elucidating their mechanisms of action in combating infections. Copaiba oil and compound **2** were found to eradicate *Trypanosoma cruzi* forms within infected macrophages through various mechanisms, modulating the immune response while directly affecting the parasites to induce their demise [88].

### 6.5. Hepatoprotective Activity

In a comprehensive investigation, the authors delved into the hepatoprotective properties of ethanol extract derived from *A. continentalis* roots against liver damage induced by *tert*-butyl hydroperoxide (t-BHP) in both Hepa1c1c7 cells and mice [89,90]. Pre-treatment with *A. continentalis* roots yielded significant reductions in liver enzyme markers, lipid peroxidation levels, and oxidative stress, while concurrently enhancing GSH levels and ameliorating liver lesions. Notably, in vitro experiments showcased the ability of *A. continentalis* roots to counteract t-BHP-induced cellular damage, enhance antioxidant enzyme activity, and trigger Nrf2 nuclear translocation through the ERK1/2 and p38 pathways. These findings suggest that the hepatoprotective effects of *A. continentalis* roots can be attributed to their antioxidant properties and modulation of antioxidant enzymes via distinct signaling pathways [89]. In a separate investigation, researchers explored the protective mechanisms of a 70% ethanol extract from *A. continentalis* roots against carbon tetrachloride (CCl_4_)-induced hepatotoxicity. Pre-treatment with *A. continentalis* root extract in mice led to significant reductions in serum ALT and AST enzymatic activities, hepatic MDA levels, and liver lesions caused by CCl_4_. Additionally, the extract prevented GSH depletion, stimulated GSH-*S*-transferase activity, and upregulated heme oxygenase-1 expression, thereby providing notable antioxidative protection. These results suggest a pivotal role for *A. continentalis* roots in alleviating acute liver injury induced by CCl_4_ through the upregulation of antioxidative proteins [90]. Moreover, a study investigated the potential of compound **2** in inhibiting lipid accumulation in a cellular model mimicking non-alcoholic fatty liver disease. HepG2 cells treated with palmitate exhibited a significant increase in intracellular triglyceride levels and the expression of lipogenic genes. However, co-administration of kaurenoic acid (**2**) effectively mitigated these effects, indicating the pharmacological potential of compound **2** in combating lipid accumulation associated with non-alcoholic fatty liver disease [91]. Another study used CCl_4_ to induce liver injury in rats and found that the 95% methanol extract of *A. continentalis* root bark had an inhibitory effect on ALT and AST activity [92].

### 6.6. Anti-Diabetic Activity

Moving on to anti-diabetic activities, it was observed that *A. continentalis* aqueous extract displayed weak *α*-glycosidase inhibitory activity (8.4%), while the methanol extract exhibited potent inhibitory activity (55.3%) [93]. Additionally, *A. continentalis* aqueous extract demonstrated a *β*-secretase inhibitory effect of 60.0%, whereas the methanol extract showed 46.1%, indicating the potential efficacy of *A. continentalis* in modulating *β*-secretase activity [94]. Furthermore, a study focused on five active diterpenoids, including *ent*-pimara-8(14),15-diene-19-oic acid, 7*β*-hydroxy-*ent*-pimara-8(14),15-diene-19-oic acid (**21**), *ent*-pimara-8(14),15-diene-19-ol (**29**), and 8*α*-hydroxy-*ent*-pimara-15-en-19-ol (**11**) and their enzyme kinetic assays. These compounds exhibited promising inhibitory effects against PTP1B, a protein tyrosine phosphatase, showcasing their potential in increasing insulin receptor phosphorylation and ameliorating hyperglycemia [95]. Aldose reductase (AR) is a key enzyme in the polyol pathway, converting glucose to sorbitol in an NADPH-dependent reaction. High glucose levels lead to excess sorbitol, causing metabolic imbalances and promoting diabetes complications. Rodriguez et al. explored the AR inhibitory activity of *A*. *continentalis* roots. Ethanol extracts and fractions were tested on rat lens homogenate. The ethanol extract showed low inhibitory activity, but the ethyl acetate fraction was most active (IC_50_, 7.76 µg/mL), though less so than the positive control TMG (IC_50_, 0.30 µg/mL). The chloroform fraction also showed low activity, while others were inactive [96]. Previous reports indicate diterpenoids, flavonoids, and triterpenoid saponins as possible AR inhibitors with anti-diabetic activity [127,128]. Further studies are needed to identify the active compounds and develop potential treatments for diabetes and related issues.

### 6.7. Cytotoxicity

Research has assessed the potential anticancer properties and underlying mechanisms of action of 70% ethanol extracts from *A. continentalis* roots on human colorectal cancer cells. Park and colleagues revealed that *A. continentalis* root extract effectively inhibited the proliferation of HCT116 and SW480 colorectal cancer cells. Specifically, it was observed that the extract significantly reduced the expression of cyclin D1 at the protein level compared to the mRNA level, indicating potential post-transcriptional regulatory mechanisms at play. Furthermore, the downregulation of cyclin D1 induced by *A. continentalis* root extract was found to be linked to proteasome activity, as evidenced by the attenuation of this effect in the presence of MG132. Interestingly, the degradation of cyclin D1 was shown to be dependent on GSK3*β* activity rather than ERK1/2 or p38 signaling pathways. Moreover, the phosphorylation of cyclin D1 at T286 induced by *A. continentalis* root extract was found to be regulated by GSK3*β*, where the inhibition of GSK3*β* activity led to a reduction in this phosphorylation event. Altogether, these findings suggest that *A. continentalis* root may target cyclin D1 for degradation via GSK3*β* [97], indicating its potential as a chemopreventive or therapeutic agent for colorectal cancer. In a separate study, *A. continentalis* leaves exhibited promising results, with cell viabilities of 17.07% and 26.79% observed on LNCaP and DU145 prostate cancer cell lines, respectively, while the stems displayed viabilities of 30.37% and 59.84% [98]. These findings suggest that *A. continentalis* leaves may hold potential in the treatment of androgen-insensitive disorders such as advanced prostate cancer. Additionally, research has shown that the ethyl acetate fraction of methanol extract from *A. continentalis* roots possesses inhibitory effects on various human cancer cells, including the induction of apoptosis in HL-60 cells. The IC_50_ values for the ethyl acetate fraction against HL-60, HepG2, HeLa, DU-145, and HT-29 cells were determined to be 56.3, 87.2, 93.2, 135.5, and 135.8 mg/mL, respectively, with no anti-proliferative effects observed on normal lymphocytes. Further analysis using DAPI staining, flow cytometry, and Western blot techniques confirmed that the ethyl acetate fraction induced apoptosis in HL-60 cells through caspase-3-mediated PARP cleavage [99].

Moreover, *A. continentalis* has shown potential in targeting B-lymphoma cells through its major diterpenoid, continentalic acid (**1**), which selectively affects cancer cells while sparing normal cells. Compound **1** was found to decrease the expression of pro-survival proteins, disrupt mitochondrial function, and activate cell death pathways. Notably, it was shown to enhance the anticancer effects of roflumilast, a drug under investigation for the treatment of B-cell malignancies [100]. These findings suggest that continentalic acid holds promise in the therapeutic management of B-cell lymphoma. Subsequent investigations into compound **1**‘s effects on human leukemia HL-60 cells and mouse fibroblast NIH 3T3 cells revealed its ability to inhibit HL-60 cell growth without affecting HaCaT keratinocytes [101]. In mouse fibroblasts, compound **1** treatment led to an increase in apoptotic cells in a time- and dose-dependent manner, mediated by the activation of caspase-3, Bak, and Bax, as well as the downregulation of Bcl-2. These results suggest that compound **1** effectively induces apoptosis in human leukemia cells [101]. The research of Kwon et al. investigated the impact of compound **1** on the proliferation and apoptosis of HepG2 cells. The MTT assay demonstrated a progressive increase in the inhibitory effect of continentalic acid over 24, 48, and 72 h, reaching its peak at 72 h. Treatment with continentalic acid led to DNA fragmentation and a dose-dependent rise in apoptotic cells. The process of apoptosis includes the activation of caspase-3, Bak, and Bax, the cleavage of PARP, and the reduction of Bcl-2. These findings suggest that compound **1** has the potential to be a valuable candidate for chemotherapy [102]. The induction of phase II enzymes, specifically quinone reductase (QR) activity, is a key chemopreventive mechanism. *A. continentalis* extracts were tested on murine hepatoma cells, revealing QR induction levels of up to 401.9%. This activity exceeded known QR inducers like t-butylhydroquinone and *β*-naphthoflavone, while exhibiting low cytotoxicity [103].

### 6.8. Other Pharmacological Effects

In another study, the effects of *A. continentalis* root on blood pressure and blood components were observed in twenty-four hypertensive elderly patients. The participants were divided into three groups: those receiving antihypertensive medications (Group A), those on medication supplemented with the root extract (Group B), and those consuming only the extract (Group C). Group C exhibited lower systolic and diastolic blood pressure compared to Groups A and B. Blood glucose levels were lower in Group C, while triglycerides increased in Group A and decreased in Group B. Additionally, total cholesterol and LDL cholesterol decreased in Groups B and C, with higher HDL cholesterol levels in Group C. Liver function markers such as GOT, GPT, and total bilirubin were higher in Group C. The root extract did not have a significant effect on blood protein or non-protein nitrogen levels. In conclusion, the consumption of the extract contributed to reduced blood pressure, blood glucose, total cholesterol, and LDL cholesterol concentrations [104]. Furthermore, an additional study examined the impact of diterpenoid acids, including continentalic acid (**1**) and kaurenoic acid (**2**), on MDA production by rat platelets in response to thrombin stimulation. Both compounds showed inhibitory effects on MDA generation [105]. Notably, compared to the control group, the 10 mM continentalic acid-treated group exhibited accelerated wound closure [106], indicating its potential to promote skin cell migration, a critical step in wound healing. Additionally, Yoon et al. investigated the sleep-enhancing properties and mechanisms of *A. continentalis* root using pentobarbital-induced sleep tests and polysomnographic recordings. A 70% ethanol extract of *A. continentalis* root was found to expedite the onset and prolong the duration of non-rapid eye movement sleep, thereby reducing wakefulness. Electroencephalogram power density assessments revealed no significant alterations in sleep quality. Moreover, the root extract was observed to enhance GABAergic synaptic transmission by modulating GABAA receptor function, independent of benzodiazepine receptors. In summary, the 70% ethanol extract of *A. continentalis* root extended the duration of non-rapid eye movement sleep without affecting its intensity [107]. Lastly, a study assessing the acute toxicity of aqueous extracts of *A. continentalis* root bark in mice was conducted. When orally administered to ICR mice at 2000 mg/kg, the extract resulted in no mortality, clinical signs, body weight fluctuations, or gross abnormalities, except for lymph node hypertrophy in male mice. No *A. continentalis*-associated changes in organ weight or histopathology were observed. The LD₅₀ and approximate LD values of *A. continentalis* extracts in mice exceeded 2000 mg/kg, indicating the non-toxic nature of these extracts [108].

## 7. Discussion

We extensively reviewed the traditional medicinal uses, geographical prevalence, botanical characteristics, phytochemical composition, and pharmacological studies of *A. continentalis*, a plant traditionally used for its medicinal properties. A total of one hundred and fifty-nine compounds are reported, highlighting that *A. continentalis* is rich in phytochemicals such as diterpenoids, steroids, triterpenoids, volatile components, phenolics, vitamins, trace elements, and other compounds. Additionally, we examined the various pharmacological studies conducted on these compounds and extracts of *A. continentalis*. A comprehensive literature analysis revealed that *A. continentalis* possesses remarkable anti-inflammatory and analgesic properties. Moreover, it holds promise in applications requiring antioxidant capabilities, antimicrobial activity, insecticidal qualities, hepatoprotective effects, anti-diabetic potential, cytotoxic properties, and other pharmacological benefits. Specifically, diterpenoids, steroids, triterpenoids, volatile components, and phenolics, which are identified as the key components mediating these pharmacological effects, have undergone extensive research and been reported multiple times.

The root of *A. continentalis* has been lauded for its medicinal qualities for an extended period, and it holds a prominent position in the literature pertaining to Chinese herbal remedies and Korean medicine. Consequently, significant scholarly research has focused on uncovering the chemical composition of *A. continentalis*, particularly the diterpenoids, a class of compounds unique to the *Aralia* genus. A total of thirty diterpenoids have been discovered in *A. continentalis*, and exploring their biological effects holds great potential for future research endeavors. This exploration could lead to the discovery of safe and effective compounds with therapeutic applications. In our previous research, we found that the fresh material of *A. continentalis* root contains 0.6% (*v*/*w*) volatile oil, with the primary component, *α*-pinene, constituting a substantial 65.0% of this oil. This chemical composition gives the volatile oil a distinct aroma and imparts various biological activities such as antibacterial, antioxidant, and anti-diabetic properties [25]. Being a natural product, the volatile oil exhibits a diverse chemical composition and significant biological activities, suggesting vast potential for applications in medicine, cosmetics, and food additives. With advancements in technology and further research, we anticipate that the volatile oil of *A. continentalis* will assume an increasingly critical role across diverse fields. We eagerly look forward to engaging more researchers in this area to collectively investigate and utilize the volatile oil of *A. continentalis*.

Furthermore, extensive research has revealed a diverse array of pharmacological effects demonstrated by *A. continentalis*, particularly highlighting its anti-inflammatory and analgesic properties, antioxidant capabilities, and antimicrobial activity. However, it is crucial to acknowledge that some studies have solely relied on extracts of *A. continentalis* rather than isolated pure compounds. Additionally, the exploration of *A. continentalis’* pharmacological effects remains incomplete, with numerous underlying mechanisms yet to be fully elucidated. Simultaneously, there is a notable dearth of in vivo research in this domain. Therefore, further investigations focusing on both in vivo studies and the mechanism of action are imperative for a comprehensive understanding of *A. continentalis*.

In summary, it is crucial to explore several key research paths concerning *A. continentalis*. Firstly, conducting a more thorough investigation into its chemical composition is necessary to identify the specific compounds responsible for its pharmacological effects. Secondly, while some components have shown promising pharmacological properties and cytotoxicity in cellular studies, further confirmation through testing on animal models is vital. Subsequently, a detailed examination of its pharmacological mechanisms is needed to provide theoretical guidance and technical support for drug development and clinical use. Lastly, there should be a focus on researching and developing volatile oils from *A. continentalis* to hasten their potential applications in medicine, cosmetics, and food additives.

## 8. Conclusions

In spite of the plethora of research findings available, there remains a notable absence of a comprehensive review concerning the traditional uses, geographical distribution, botanical description, phytochemistry, and pharmacology of *A. continentalis*. As a result, the primary objective of this review was to conduct a thorough examination of the existing literature on *A. continentalis* by utilizing various databases to explore these specific aspects extensively. Furthermore, the goal of this review was to identify potential opportunities for future research endeavors, including the isolation and characterization of novel compounds found in *A. continentalis*, in-depth pharmacological evaluations, and the elucidation of its underlying mechanisms of action, with a particular focus on the research and development of volatile oils. The outcomes of this investigation are anticipated to establish a solid groundwork for the extraction and identification of constituents, the formulation of products, and the clinical application of *A. continentalis*.

## Figures and Tables

**Table 1:** Diterpenoids isolated or identified from *Aralia continentalis*.

No.	Name	Formula	Exact Theoretical M. W.	Source	Characterization Method	Ref.
1.	Continentalic acid	C_20_H_30_O_2_	302.2246	roots	EI-MS, ^1^H NMR, ^13^C NMR	[1]
				roots	^1^H NMR, ^13^C NMR	[2]
				roots	ESI-MS, ^1^H NMR, ^13^C NMR	[3]
				roots	IR, TLC, ESI-MS, ^1^H NMR, ^13^C NMR, ^1^H-^1^H COSY, HMBC, HMQC, ${\left[\alpha \right]}_{D}^{25}$	[4]
				roots	mp, ESI-MS, ^1^H NMR, ^13^C NMR, HMBC, ${\left[\alpha \right]}_{D}^{20}$	[5]
				roots	mp, UV, ^1^H NMR, ^13^C NMR, ${\left[\alpha \right]}_{D}^{20}$	[6]
				roots	mp, UV, IR, MS, ^1^H NMR, ^13^C NMR, ${\left[\alpha \right]}_{D}^{25}$	[7]
				roots	EI-MS, ^1^H NMR, ^13^C NMR	[1]
				roots	^1^H NMR, ^13^C NMR	[8]
				roots	ESI-MS, ^1^H NMR, ^13^C NMR	[9]
2.	Kaurenoic acid	C_20_H_30_O_2_	302.2246	roots	^1^H NMR, ^13^C NMR	[2]
				roots	EI-MS, ^1^H NMR, ^13^C NMR	[1]
				roots	Chemical reaction, TLC, mp, IR, GC	[10]
				roots	Chemical reaction, elemental analysis, IR, EI-MS, ^1^H NMR, ^13^C NMR, ${\left[\alpha \right]}_{D}^{20}$	[11]
				roots	^1^H NMR, ^13^C NMR	[12]
				roots	^1^H NMR, ^13^C NMR	[13]
3.	Acanthoic acid	C_20_H_30_O_2_	302.2246	roots	^1^H NMR, ^13^C NMR	[2]
4.	Continentanol	C_19_H_30_O	274.2297	roots	UV, ESI-MS, ^1^H NMR, ^13^C NMR, 2D NMR	[14]
				roots	IR, EI-MS, HRESIMS, ^1^H NMR, ^13^C NMR, HMBC, ^1^H-^1^H COSY, NOESY, ${\left[\alpha \right]}_{D}^{25}$	[9]
5.	*Ent-*pimara-8(14),15-diene-19-ol	C_20_H_32_O	288.2453	roots	EI-MS, ^1^H NMR, ^13^C NMR	[1]
6.	7-oxo-*ent*-pimara-8(14),15-diene-19-oic acid	C_20_H_28_O_3_	316.2038	roots	EI-MS, ^1^H NMR, ^13^C NMR	[1]
				roots	^1^H NMR, ^13^C NMR	[8]
7.	16-Hydroxy-17-isovaleroyloxy*ent*-kauran-19-oic acid	C_25_H_40_O_5_	420.2876	roots	EI-MS, ^1^H NMR, ^13^C NMR	[1]
8.	17-Hydroxy-*ent*-kaur-15-en-19-oic acid	C_20_H_30_O_3_	318.2195	roots	EI-MS, ^1^H NMR, ^13^C NMR	[1]
9.	15,16-Epoxy-17-hydroxy-*ent*-kauran-19-oic acid	C_20_H_30_O_4_	334.2144	roots	EI-MS, ^1^H NMR, ^13^C NMR	[1]
				roots	^1^H NMR, ^13^C NMR	[8]
10.	16*α*,17-Dihydroxy-*ent*-kauran-19-oic acid	C_20_H_32_O_4_	336.2301	roots	EI-MS, ^1^H NMR, ^13^C NMR	[1]
11.	8*α*-Hydroxy-*ent*-pimara-15-en-19-ol	C_20_H_34_O_2_	306.2559	roots	EI-MS, ^1^H NMR, ^13^C NMR	[1]
12.	4-*epi*-Rulopezol	C_19_H_30_O	274.2297	roots	EI-MS, ^1^H NMR, ^13^C NMR	[1]
13.	4*β*-Hydroxy-19-nor-(-)-pimara-8(14),15-diene	C_19_H_30_O	274.2297	roots	EI-MS, ^1^H NMR, ^13^C NMR	[1]
14.	*Ent*-kaur-16-en-19-oic acid	C_20_H_30_O_2_	302.2246	roots	Chemical reaction, ^1^H NMR, ^13^C NMR	[15]
				roots	^1^H NMR, ^13^C NMR	[8]
				roots	^1^H NMR, ^13^C NMR	[13]
				roots	Chemical reaction, ^1^H NMR, ^13^C NMR	[16]
15.	*Ent*-pimar-15-en-8*β*,19-diol	C_20_H_34_O_2_	306.2559	roots	Chemical reaction, ^1^H NMR, ^13^C NMR	[15]
				roots	Chemical reaction, ^1^H NMR, ^13^C NMR	[16]
16.	16*α*-Hydroxy-*ent*-kauran-19-oic acid	C_20_H_32_O_3_	320.2351	roots	Chemical reaction, ^1^H NMR, ^13^C NMR	[16]
17.	13*β*-Hydroxy-7-oxoabiet-8(14)-en-19,6*β*-olide	C_20_H_28_O_4_	332.1988	roots	Chemical reaction, ^1^H NMR, ^13^C NMR	[16]
18.	*Ent*-16*β*,17-dihydroxy-kauran-19-oic acid	C_20_H_32_O_4_	336.2301	roots	Chemical reaction, ^1^H NMR, ^13^C NMR	[16]
19.	*Ent*-pimara-8(14),15-dien-19-oic acid	C_20_H_30_O_2_	302.2246	roots	Chemical reaction, TLC, mp, IR, GC	[10]
20.	16*α*-Hydroxy-*ent*-kauran-19-oic acid	C_20_H_32_O_3_	320.2351	roots	Chemical reaction, elemental analysis, IR, MS, ^1^H NMR, ^13^C NMR, ${\left[\alpha \right]}_{D}^{25}$	[11]
21.	7*β*-Hydroxy-*ent*-pimara-8(14),15-diene-19-oic acid	C_20_H_30_O_3_	318.2195	roots	^1^H NMR, ^13^C NMR	[8]
22.	16*α*H,17-Isovaleryloxy-*ent*-kauran-19-oic acid	C_25_H_40_O_4_	404.2927	roots	ESI-MS, ^1^H NMR, ^13^C NMR	[9]
23.	Melanocane A	C_20_H_32_O_4_	336.2301	roots	ESI-MS, ^1^H NMR, ^13^C NMR	[9]
24.	*Ent*-17-hydroxy-kaur-15-en-19-oic acid	C_20_H_30_O_3_	318.2195	roots	ESI-MS, ^1^H NMR, ^13^C NMR	[9]
25.	*trans*-Communol	C_20_H_32_O	288.2453	roots	ESI-MS, ^1^H NMR, ^13^C NMR	[9]
26.	18-Nor-*ent*-pimara-8(14),15-diene-4*β*-ol	C_19_H_30_O	274.2297	roots	ESI-MS, ^1^H NMR, ^13^C NMR	[9]
27.	4-Epiruilopeziol	C_19_H_30_O	274.2297	roots	ESI-MS, ^1^H NMR, ^13^C NMR	[9]
28.	*Ent*-continentalic acid	C_20_H_30_O_2_	302.2246	roots	ESI-MS, ^1^H NMR, ^13^C NMR	[9]
				roots	^1^H NMR, ^13^C NMR	[12]
29.	*Ent*-pimara-8(14),15-diene-19-ol	C_20_H_32_O	288.2453	roots	ESI-MS, ^1^H NMR, ^13^C NMR	[9]
30.	3*α*,16*α*-dihydroxukaurane-17-*O*-*β*-*D*-glucoside	C_26_H_44_O_8_	484.3036	roots	Chemical reaction, ^1^H NMR, ^13^C NMR	[15]

UV: Ultraviolet spectrophotometry; mp: Melting point; IR: Infrared spectroscopy; TLC: Thin-layer chromatography; GC: Gas chromatography; MS: Mass spectrometry; EI-MS: Electron impact mass spectrometry; ESI-MS: Electrospray ionization mass spectrometry; ^1^H NMR: Hydrogen-1 nuclear magnetic resonance spectrometry; ^13^C-NMR: Carbon-13 nuclear magnetic resonance spectrometry; COSY: Correlation spectroscopy; HMBC: ^1^H Detected heteronuclear multiple-bond correlation; HMQC: ^1^H Detected heteronuclear multiple-quantum coherence; 2D NMR: Two-dimensional nuclear magnetic resonance spectrometry; HRESIMS: High-resolution electrospray ionization mass spectrometry; NOESY: Nuclear overhauser effect spectroscopy.

**Table 2:** Steroids and triterpenoids isolated or identified from *Aralia continentalis*.

No.	Name	Formula	Exact Theoretical M. W.	Source	Characterization Method	Ref.
31.	Stigmasterol	C_29_H_48_O	412.3705	roots	IR, TLC, ESI-MS, ^1^H NMR, ^13^C NMR, ^1^H-^1^H COSY, HMBC, HMQC, ${\left[\alpha \right]}_{D}^{25}$	[4]
				roots	mp, UV, ^1^H NMR, ^13^C NMR, ${\left[\alpha \right]}_{D}^{20}$	[6]
				roots	Chemical reaction, mp, ^1^H NMR, ^13^C NMR	[15]
				roots	Chemical reaction, ^1^H NMR, ^13^C NMR	[16]
				roots	ESI-MS, ^1^H NMR, ^13^C NMR	[9]
				roots	mp, ^1^H NMR, ^13^C NMR, HMBC, ${\left[\alpha \right]}_{D}^{25}$	[17]
32.	*β*-Sitosterol	C_29_H_50_O	414.3862	roots	mp, TLC, IR, ${\left[\alpha \right]}_{D}^{25}$	[11]
				roots	ESI-MS, ^1^H NMR, ^13^C NMR	[9]
33.	Oleanolic acid	C_30_H_48_O_3_	456.3603	roots	Chemical reaction, mp, ^1^H NMR, ^13^C NMR	[15]
				roots	HPLC	[18]
				roots	mp, IR	[19]
34.	Ursolic acid	C_30_H_48_O_3_	456.3603	roots	Chemical reaction, ^1^H NMR, ^13^C NMR	[15]
35.	Daucosterol	C_35_H_60_O_6_	576.4390	roots	Chemical reaction, ^1^H NMR, ^13^C NMR	[16]
				roots	Chemical reaction, elemental analysis, IR, EI-MS, FD-MS, ^13^C NMR, ${\left[\alpha \right]}_{D}^{20}$	[11]
36.	Oleanolic acid 28-*O*-*β*-*D*-glucopyranosyl ester	C_36_H_58_O_8_	618.4132	aerial parts	mp, IR, ^1^H NMR, ^13^C NMR	[20]
37.	Hederagenin 28-*O*-*β*-*D*-glucopyranosyl ester	C_36_H_58_O_9_	634.4081	aerial parts	mp, IR, ^1^H NMR, ^13^C NMR	[20]
38.	Chikusetsusaponin IVa	C_42_H_66_O_14_	794.4453	aerial parts	mp, IR, ^1^H NMR, ^13^C NMR	[20]
39.	Udosaponin A	C_41_H_64_O_13_	764.4347	aerial parts	mp, IR, ^1^H NMR, ^13^C NMR	[20]
40.	Salsoloside C	C_47_H_74_O_18_	926.4875	aerial parts	mp, IR, ^1^H NMR, ^13^C NMR	[20]
41.	Salsoloside C methyl ester	C_48_H_76_O_18_	940.5032	leaves	mp, IR, MS, ^1^H NMR, ^13^C NMR	[21]
42.	Udosaponin F	C_48_H_76_O_20_	972.4930	aerial parts	mp, IR, ^1^H NMR, ^13^C NMR	[20]
43.	Udosaponin C	C_53_H_84_O_23_	1088.5403	aerial parts	Chemical reaction, mp, IR, ^1^H NMR, ^13^C NMR	[20]
44.	Congmuyenoside A	C_48_H_78_O_19_	958.5137	leaves	Chemical reaction, ^1^H NMR, ^13^C NMR	[22]
45.	Congmuyanoside C	C_42_H_68_O_14_	796.4609	tender buds	Chemical reaction, ^1^H NMR, ^13^C NMR	[23]

UV: Ultraviolet spectrophotometry; mp: Melting point; IR: Infrared spectroscopy; TLC: Thin-layer chromatography; HPLC: High-performance liquid chromatography; EI-MS: Electron impact mass spectrometry; FD-MS: Field desorption–mass spectrometry; ESI-MS: Electrospray ionization mass spectrometry; ^1^H NMR: Hydrogen-1 nuclear magnetic resonance spectrometry; ^13^C-NMR: Carbon-13 nuclear magnetic resonance spectrometry; COSY: Correlation spectroscopy; HMBC: ^1^H Detected heteronuclear multiple-bond correlation; HMQC: ^1^H Detected heteronuclear multiple-quantum coherence.

**Table 3:** Volatile components isolated or identified from *Aralia continentalis*.

No.	Name	Formula	Exact Theoretical M. W.	Source	Characterization Method	Ref.
46.	*α*-Pinene	C_10_H_16_	136.1252	roots	GC-MS	[24]
				roots	GC-MS	[25]
47.	*β*-Pinene	C_10_H_16_	136.1252	roots	GC-MS	[24]
				roots	GC-MS	[25]
48.	8-Nonynoic acid	C_9_H_14_O_2_	154.0994	roots	GC-MS	[24]
49.	2,5-Dimethyl-3-methylene-1,5-heptadiene	C_10_H_16_	136.1252	roots	GC-MS	[24]
50.	3,7,11-Trimethyl-1,3,6,10-dodecatetraene	C_15_H_24_	204.1878	roots	GC-MS	[24]
51.	8-(1-Methylethylidene)bicyclo [5.1.0]octane	C_11_H_18_	150.1409	roots	GC-MS	[24]
52.	1-Dodecyne	C_12_H_22_	166.1722	roots	GC-MS	[24]
53.	2,5-Dimethyl-1,5-cyclooctadiene	C_10_H_16_	136.1252	roots	GC-MS	[24]
54.	*β*-Caryophyllene	C_15_H_24_	204.1878	roots	GC-MS	[24]
55.	a-Gurjunene	C_15_H_24_	204.1878	roots	GC-MS	[24]
56.	Copaene	C_15_H_24_	204.1878	roots	GC-MS	[24]
				roots	GC-MS	[25]
57.	*α*-Cadinene	C_15_H_24_	204.1878	roots	GC-MS	[24]
58.	1H-3a,7-Methanoazulene, octahydro-1,4,9,9-tetramethyl-	C_15_H_26_	206.2035	roots	GC-MS	[24]
59.	Spathulene	C_15_H_22_	202.1722	roots	GC-MS	[24]
60.	Linalool	C_10_H_18_O	154.1358	roots	GC-MS	[24]
61.	12,15-Octadecadienoic acid methyl ester	C_19_H_34_O_2_	294.2559	roots	GC-MS	[24]
62.	Oleic acid	C_18_H_34_O_2_	282.2559	seeds	GC-MS	[26]
63.	Petroselinic acid	C_18_H_34_O_2_	282.2559	seeds	GC-MS, IR, ^1^H NMR, ^13^C NMR, HMQC	[26]
64.	Linoleic acid	C_18_H_32_O_2_	280.2402	seeds	GC-MS	[26]
65.	Palmitic acid	C_16_H_32_O_2_	256.2402	seeds	GC-MS	[26]
66.	11-Hexacosenoic acid	C_26_H_50_O_2_	394.3811	roots	Chemical reaction, ^1^H NMR, ^13^C NMR	[15]
67.	*n*-Hexadecanoic acid methyl ester	C_17_H_34_O_2_	270.2559	roots	Chemical reaction, ^1^H NMR, ^13^C NMR	[16]
68.	Sabinene	C_10_H_16_	136.1252	roots	GC-MS	[25]
69.	Naphthalene, 1,2-dihydro-1,1,6-trimethyl-	C_13_H_16_	172.1252	roots	GC-MS	[25]
70.	*α*-Ylangene	C_15_H_24_	204.1878	roots	GC-MS	[25]
71	*β*-Longipinene	C_15_H_24_	204.1878	roots	GC-MS	[25]
72.	1,2,9,10-Tetradehydroaristolane	C_15_H_22_	202.1722	roots	GC-MS	[25]
73.	(+)-*β*-Caryophyllene	C_15_H_24_	204.1878	roots	GC-MS	[25]
74.	(+)-*α*-Amorphene	C_15_H_24_	204.1878	roots	GC-MS	[25]
75.	*γ*-Himachalene	C_15_H_24_	204.1878	roots	GC-MS	[25]
76.	Germacrene D	C_15_H_24_	204.1878	roots	GC-MS	[25]
77.	*β*-Guaiene	C_15_H_24_	204.1878	roots	GC-MS	[25]
78.	(-)-Nootkatene	C_15_H_22_	202.1722	roots	GC-MS	[25]
79.	*cis*-Calamenene	C_15_H_22_	202.1722	roots	GC-MS	[25]
80.	*α*-Copaen-11-ol	C_15_H_24_O	220.1827	roots	GC-MS	[25]
81.	*α*-Calacorene	C_15_H_20_	200.1565	roots	GC-MS	[25]
82.	*β*-Vetivenene	C_15_H_22_	202.1722	roots	GC-MS	[25]
83.	*α*-Agarofuran	C_15_H_24_O	220.1827	roots	GC-MS	[25]
84.	(-)-Globulol	C_15_H_26_O	222.1984	roots	GC-MS	[25]
85.	Cadalin	C_15_H_18_	198.1409	roots	GC-MS	[25]
86.	T-Cadinol	C_15_H_26_O	222.1984	roots	GC-MS	[25]
87.	*α*-Acorenol	C_15_H_26_O	222.1984	roots	GC-MS	[25]
88.	*β*-Eudesmol	C_15_H_26_O	222.1984	roots	GC-MS	[25]
89.	8a-Isopropyl-3-methyl-1,2,4,5,8,8a-hexahydroazulene-6-carbaldehyde	C_15_H_22_O	218.1671	roots	GC-MS	[25]
90.	4(15),5,10(14)-Germacratrien-1-ol	C_15_H_24_O	220.1827	roots	GC-MS	[25]
91.	14-Hydroxy-*α*-muurolene	C_15_H_24_O	220.1827	roots	GC-MS	[25]
92.	3-Desoxyisopetasol	C_15_H_22_O	218.1671	roots	GC-MS	[25]
93.	Falcarinol	C_17_H_24_O	244.1827	roots	GC-MS	[25]
94.	Methyl 5,7-hexadecadiynoate	C_17_H_26_O_2_	262.1933	roots	GC-MS	[25]

IR: Infrared spectroscopy; GC-MS: Gas chromatography–mass spectrometry; ^1^H NMR: Hydrogen-1 nuclear magnetic resonance spectrometry; ^13^C-NMR: Carbon-13 nuclear magnetic resonance spectrometry; HMQC: ^1^H Detected heteronuclear multiple-quantum coherence.

**Table 5:** Vitamins and trace elements isolated or identified from *Aralia continentalis*.

No.	Name	Formula	Exact Theoretical M. W.	Source	Characterization Method	Ref.
119.	Calcium	Ca	39.9626	roots, leaves	ICP-AES, atomic absorption spectrophotometer	[30]
120.	Phosphorus	P	30.9738	roots, leaves	ICP-AES, atomic absorption spectrophotometer	[30]
121.	Ferrum	Fe	55.9349	roots, leaves	ICP-AES, atomic absorption spectrophotometer	[30]
122.	Natrium	Na	22.9898	roots, leaves	ICP-AES, atomic absorption spectrophotometer	[30]
123.	Potassium	K	38.9637	roots, leaves	ICP-AES, atomic absorption spectrophotometer	[30]
124.	Magnesium	Mg	23.9850	roots, leaves	ICP-AES, atomic absorption spectrophotometer	[30]
125.	Zinc	Zn	63.9291	roots, leaves	ICP-AES, atomic absorption spectrophotometer	[30]
126.	Retinol	C_20_H_30_O	286.2297	roots, leaves	HPLC	[30]
127.	*β*-Carotene	C_40_H_56_	536.4382	roots, leaves	HPLC	[30]
				leaves	LC-SIM-MS	[31]
128.	Thiamin	C_12_H_17_ClN_4_OS	300.0812	roots, leaves	HPLC	[30]
129.	Riboflavin	C_17_H_20_N_4_O_6_	376.1383	roots, leaves	HPLC	[30]
130.	Niacin	C_6_H_5_NO_2_	123.0320	roots, leaves	HPLC	[30]
				roots	EI-MS, ^1^H NMR, ^13^C NMR	[1]
131.	Ascorbic acid	C_6_H_8_O_6_	176.0321	roots, leaves	HPLC	[30]
132.	Violaxanthin	C_40_H_56_O_4_	600.4179	leaves	LC-SIM-MS	[31]
133.	Neoxanthin	C_40_H_56_O_4_	600.4179	leaves	LC-SIM-MS	[31]
134.	Luteoxanthin	C_40_H_56_O_4_	600.4179	leaves	LC-SIM-MS	[31]
135.	Lutein	C_40_H_56_O_2_	568.4280	leaves	LC-SIM-MS	[31]
136.	Zeaxanthin	C_40_H_56_O_2_	568.4280	leaves	LC-SIM-MS	[31]

ICP-AES: Inductively coupled plasma-atomic emission spectrometry; HPLC: High-performance liquid chromatography; EI-MS: Electron impact mass spectrometry; LC-SIM-MS: Liquid chromatography–SIM–mass spectrometry; ^1^H NMR: Hydrogen-1 nuclear magnetic resonance spectrometry; ^13^C-NMR: Carbon-13 nuclear magnetic resonance spectrometry.

## Data Availability

Data are contained within the article.
